# Factors affecting aerosol SARS-CoV-2 transmission via HVAC systems; a modeling study

**DOI:** 10.1371/journal.pcbi.1009474

**Published:** 2021-10-18

**Authors:** Zachary J. Cotman, Michael J. Bowden, Barrett P. Richter, Joseph H. Phelps, Christopher J. Dibble

**Affiliations:** Battelle Memorial Institute, Columbus, Ohio, United States of America; Stanford University, UNITED STATES

## Abstract

The role of heating, ventilation, and air-conditioning (HVAC) systems in the transmission of SARS-CoV-2 is unclear. To address this gap, we simulated the release of SARS-CoV-2 in a multistory office building and three social gathering settings (bar/restaurant, nightclub, wedding venue) using a well-mixed, multi-zone building model similar to those used by Wells, Riley, and others. We varied key factors of HVAC systems, such as the Air Changes Per Hour rate (ACH), Fraction of Outside Air (FOA), and Minimum Efficiency Reporting Values (MERV) to examine their effect on viral transmission, and additionally simulated the protective effects of in-unit ultraviolet light decontamination (UVC) and separate in-room air filtration. In all building types, increasing the ACH reduced simulated infections, and the effects were seen even with low aerosol emission rates. However, the benefits of increasing the fraction of outside air and filter efficiency rating were greatest when the aerosol emission rate was high. UVC filtration improved the performance of typical HVAC systems. In-room filtration in an office setting similarly reduced overall infections but worked better when placed in every room. Overall, we found little evidence that HVAC systems facilitate SARS-CoV-2 transmission; most infections in the simulated office occurred near the emission source, with some infections in individuals temporarily visiting the release zone. HVAC systems only increased infections in one scenario involving a marginal increase in airflow in a poorly ventilated space, which slightly increased the likelihood of transmission outside the release zone. We found that improving air circulation rates, increasing filter MERV rating, increasing the fraction of outside air, and applying UVC radiation and in-room filtration may reduce SARS-CoV-2 transmission indoors. However, these mitigation measures are unlikely to provide a protective benefit unless SARS-CoV-2 aerosol emission rates are high (>1,000 Plaque-forming units (PFU) / min).

## Author summary

Evidence of heating, ventilation, and air-conditioning (HVAC) systems facilitating SARS-CoV-2 transmission is limited, and we do not know the protective effects of recommended HVAC modifications. To address these gaps, we simulated HVAC systems in an office building and three social settings (bar/restaurant, nightclub, and wedding venue), varying key parameters to identify those associated with SARS-CoV-2 transmission. Ultimately, we found that HVAC systems are unlikely to facilitate spread, and that most common mitigation strategies, such as increasing air circulation rates, filtration efficiency, or the fraction of outside air, are able to reduce transmission rates. However, the importance of these mitigation measures is negligible in commercial settings when few SARS-CoV-2 particles are emitted via aerosol; only at very high emission rates did we see substantial reductions in transmission. Our results also indicated that HVAC systems cannot reduce transmission risk to zero. Validation efforts suggested that protective HVAC measures (e.g., increasing air circulation rates, using UVC (ultraviolet light in the 100–289 nm range) filters) may be more beneficial in residential settings, though this was not studied in detail. Identifying the emission rate of SARS-CoV-2 from infected individuals, as well as the dose necessary to infect humans, remains a key data gap.

## 1.0 Introduction

### 1.1 Background

There is conflicting evidence in the literature on the impact of HVAC systems on the dispersion of SARS-CoV-2 in indoor spaces [1]. HVAC systems have the potential to fuel transmission via small aerosol particles, which can remain airborne for minutes to hours [2], in contrast with larger respiratory droplets that tend to settle near to their emission source [3]. In one study, a shared ventilation shaft connecting bathrooms in an apartment complex was linked to transmission of SARS-CoV-2 between floors [4]. This example strongly suggests transmission via small aerosol particles and also shows that passive pathogen transport can occur between connected rooms, but it does not demonstrate that shared, central HVAC systems can facilitate the spread of SARS-CoV-2 when in use. In a separate study, an HVAC system was linked to SARS-CoV-2 transmission in a restaurant in Guangzhou, China [5], where low rates of ventilation (~0.7 Air Changes Per Hour [ACH]) coupled with high airflow in a particular direction were hypothesized to result in infection of individuals at several nearby tables. However, surface samples of the air conditioning unit inlets and outlets were negative for SARS-CoV-2, questioning whether it was air recirculation *per se* or simply directional air flow that facilitated transmission [5]. The study suggests that airflow from HVAC systems can potentially influence transmission by facilitating long-distance particle spread in an enclosed location. However, the restaurant study does not imply that central HVAC systems accelerate transmission to other rooms of a building.

It has been shown that SARS-CoV-2 can be found inside HVAC systems, potentially aiding spread via the aerosol route [6]. In an Oregon hospital, for instance, SARS-CoV-2 RNA was found in 25% of sampled air handling units [7]. The highest proportion of positive samples came from pre-filters (35%), while viral RNA was also detected at the final filters (16.7% of positive samples) and supply air dampers (20.8% of positive samples) [7]. Notably, supply air dampers were located past both the pre-filter (Minimum Efficiency Reporting Values-10 or simply MERV-10) and final filter (MERV-15) stages [7]. However, detection of viral RNA does not indicate that samples were infectious, because the concentration of RNA copies can be orders of magnitude higher than the concentration of infectious virus [8]. In these studies, no transmission events were attributed to aerosol transmission via the HVAC system. Other purported examples of SARS-CoV-2 transmission via HVAC systems [1], for instance on the Diamond Princess cruise ship, show conflicting evidence, with HVAC facilitation considered plausible by some [9] but unlikely by others, due to the lack of between-cabin transmission [10]. Thus, it is still unclear whether HVAC systems facilitate the aerosol spread of SARS-CoV-2.

Though limited evidence exists for HVAC facilitation in the current COVID-19 pandemic, HVAC systems have been implicated in the spread of other pathogens. In the 2002/2003 SARS-CoV-1 outbreak, hospital transmission was a significant factor in the overall burden of disease. HVAC systems were hypothesized to play a role in hospital transmission, and Li et al. studied the potential role of the HVAC system in a large SARS-CoV-1 outbreak in Hong Kong [11]. Using computational fluid dynamics models and detailed in-person measurements, the authors concluded that air distribution in a particular ward was associated with a large transmission event where over 140 individuals became infected [11]. Similarly, viable virus was detected in a hospital air handling system exhaust damper during a large hospital outbreak of Middle East Coronavirus Syndrome (MERS) in 2015, supporting the notion that spread of infectious aerosols can be impacted by HVAC systems [12].

While there is limited evidence for HVAC systems facilitating SARS-CoV-2 transmission, several groups have provided recommendations for limiting SARS-CoV-2 exposure in indoor environments. The American Society of Heating, Refrigerating and Air-Conditioning Engineers (ASHRAE) states that mechanical air filters, quantified by the MERV rating, may be able to reduce the spread of airborne viruses through an indoor environment [13], though only filters with MERV values ≥13 are able to remove individual virus particles from the air [13]. Typical residential and commercial HVAC systems use filters with MERV ratings of 5–8 [14]. HVAC experts mention that increasing MERV ratings may help reduce aerosol concentrations, but has drawbacks in terms of reducing HVAC lifespan, while ultraviolet lights, particularly those with emitted wavelengths in the 100–280 nm range (UVC) may also provide benefits [15]. UVC has been linked with reductions in aerosolized bacteria, fungi, and viruses [16–18], including SARS-CoV-2 [19]. The U.S. Federal Interagency Committee on Indoor Air Quality has also provided several recommendations for HVAC systems aimed to reduce COVID-19 transmission [20]. These include installing MERV-13 filters (if the system allows it), with MERV-14 or HEPA (high efficiency particulate air) filters (equivalent to MERV-16) preferred [20], alongside reducing air recirculation by increasing the fraction of outside air (FOA) [20]. Despite these recommendations, the impact of HVAC systems in SARS-CoV-2 transmission is unclear. Can HVAC systems facilitate SARS-CoV-2 spread? And what HVAC system attributes, if any, are effective at reducing indoor transmission?

### 1.2 Approach

To answer these questions, we simulated the release of SARS-CoV-2 aerosols in two types of indoor scenarios: a full workday in an office building and social gatherings in a bar/restaurant, wedding reception, and nightclub. We systematically modified important HVAC parameters of each building and estimated the fraction of individuals infected in each location during a typical residence time.

To simulate the movement of aerosol SARS-CoV-2 within the built environment, we created a model which uses a network of well-mixed zones and bi-directional mixing of air between zones. Aerosol release was modeled as a continuous point source emission in a specific zone, where exchanges with other zones occurred via doors, large openings, or shared HVAC ducts. In our model, air from multiple building zones is exchanged to a central or shared HVAC zone, where it is mixed, filtered, and vented, then exchanged back to serviced zones. In this way, aerosols were allowed to be transported via the HVAC system from the SARS-CoV-2 release zone to other zones in the model. Therefore, higher air exchange rates (air changes per hour, ACH) increase not only the amount of air filtered and vented, but also the total air transport between zones via the HVAC system, potentially facilitating SARS-CoV-2 spread beyond the release zone. Modeled aerosol particles were distributed into 12 bins by diameter, allowing us to explicitly account for size-based particle settling rates and mechanical HVAC system filtration efficiency. Infectious particles were removed from indoor air via exchange with outside air, settling, filtration, inactivation via UVC light, and biological decay. Section 4.3 provides additional detail on these mechanics and their dependency on aerosol particle size.

Similar approaches for modeling infection risk in indoor environments have been applied in studies of SARS-CoV-2. Buonanno et al. applied the Wells-Riley single-zone model to estimate indoor infection risk in common Italian commercial spaces, using a viral-load-emission model dependent on respiratory fluid type, droplet size, and activity level [21]. Bazant and Bush built on the same mathematical approach to study the differences in infection probability from exposure to well-mixed aerosols in different scenarios [22]. They simulated occupancy levels representative of normal conditions and those where a 6-foot rule is applied (lower occupancy), and also estimated the effect of limiting exposure time on infection risk [22]. Miller et al. used the well-mixed, single-zone approach to both explore the effects of HVAC ventilation rate, event duration, and deposition rate on infection risk, as well as estimate a SARS-CoV-2 release rate (in quanta-per-hour) for the Skagit Valley Chorale outbreak [23]. These studies all apply the well-mixed approximation to indoor zone modeling, as we do in our approach. A significant difference, however, is the use of the Wells-Riley infection probability model involving the “quanta” of infection [24] in these studies, whereas a probit dose-response model was used in our study to estimate infection probability from an exposure dose [25]. In Section 2.5, we compare our approach with that of Buonanno et al. [21] to assess similarities and differences.

People were modeled in groups and moved between modeled zones according to stochastically determined schedules. For example, in office building scenarios, people were modeled in groups of six. Each group was assigned an office zone, given an arrival time, total time in the office for work, lunch-break time, and time spent in meetings for which they were assigned other office zones to visit. At each time step, the inhaled dose of virus received by each group was updated according to the concentration in the group’s current zone. Probit dose-response modeling [25] was then applied to assess the probability of infection for each group.

Table 1 provides a list of key model parameters, their values or experimental ranges, and references. Pertinent HVAC system parameters investigated are air changes per hour (ACH), fraction of outside air (FOA), and filter efficiency (ASHRAE MERV rating). Effects of ultraviolet light (UVC) decontamination and portable in-room filtration units were also modeled. Other model behaviors or parameters, such as individual group behaviors, were modeled stochastically via Monte Carlo sampling. A complete description of model mechanics, parameters, and the probit dose-response model may be found in Section 4.0. Parameters specific to the office building and social gathering scenarios, including descriptions of population movement mechanics, may be found in Sections 4.5 and 4.6.

**Table 1:** Parameter values and ranges used in modeling transport of SARS-CoV-2 in indoor scenarios.

Parameter	Values or Range	Description
**ACH**	2, 6, 10, 20, 30* Default: scenario specific**	Air changes per hour. Number of times building air is recirculated through the HVAC system per hour. For scenario specific default values, see Table 10 and Table 11.
**MERV Filter Rating**	4, 8, 12, 16*	Minimum Efficiency Reporting Value. Efficiency rating for mechanical HVAC system filters. See Table 9 for filtration efficiency by particle size. Filtration efficiency per particle filtration bin chosen stochastically from values in Table 9 on a per-realization basis.
**FOA**	0.1, 0.3, 0.5, 0.9* Default: 0.04–1.0**	Fraction of outside air. Fraction of recirculated building air that is replaced with outdoor air.
**UVC Filtration**	0.9, 0.99*	Ultraviolet C. Fraction of viruses inactivated when exposed to UVC decontamination. See Section 4.1 for details and sources.
**In room filtration rate**	10 *m*^3^/*min*	Volume of air filtered by portable in-room units per minute.
**In room filtration efficiency**	0.99	Fraction of aerosol particles removed from air filtered by portable in-room units.
**Indoor Air Speed**	0.1–0.2** *m/s*	Speed of air movement inside the building which governs the rate of exchange between building zones. Representative of indoor air speeds associated with comfortable indoor environments using ASHRAE Standard 55 guidance [26].
**Release Rates**	100, 500, 1000, 3,000,. . ., 10,000* *PFU/min*	Number of aerosolized plaque forming units released by infected emitter per minute. See section 4.2 for details and sources.
**Aerosol particle MMAD**	4.0 *μm*	Post-evaporation dried particle mass median aerodynamic diameter [3].
**Aerosol particle GSD**	2	Geometric standard deviation of the aerosolized particle distribution (Section 4.2.3).
**Biological decay rate**	0.0088 *fraction/min*	Fraction of aerosolized particles which become inactive per minute. Average of [27,28].
**Respirable Particle Size Range**	1–10 *μm*	Diameter of particles which are modeled as depositing in the alveoli and contributing to dose (Section 4.4).
**ID**_**50**_	240 PFU	SARS-CoV-1 median infectious dose, used here as a surrogate for that of SARS-CoV-2. Used in the probit dose response model in determining probability of infection [29,30].
**Probit slope**	1.16	SARS-CoV-1 probit model slope used here as a surrogate for that of SARS-CoV-2 [29,30].

*Chosen systematically

**Chosen stochastically on a per-realization basis.

To validate whether or not the model returns reasonable infection probabilities, we designed a simple apartment building to mimic the pertinent aspects in the South Korean apartment complex outbreak as described by Hwang et al. [4]. We then used a subset of HVAC parameters appropriate for residential settings for testing. The settings used cover a range of possibilities for HVAC systems, both falling below and exceeding residential ASHRAE standards, which are generally lower than commercial standards. We simulated a three-day symptomatic aerosol release from a single infected emitter and assessed the probability of infection for residents in connected apartments. Along with HVAC settings and release rates, we also varied the time spent in connected apartments, which could impact the total dose received. Summary results are presented in Section 2.4. A full description of both the event and the model designed for validation may be found in Appendix C in S1 Text.

## 2.0 Results

Overall, we found that increasing the ACH was the most impactful mitigation measure across all scenarios and the only one to show meaningful efficacy at low aerosol emission rates (<1,000 PFU / min). Increasing the FOA or filter MERV rating also reduced infections, but primarily at higher emission rates. UVC and in-room filters appear effective in our model. Our results found very little evidence that HVAC systems facilitate SARS-CoV-2 spread, and most infections occurred in individuals who spent time in the simulated release zone.

### 2.1 Office scenario results

**Fig 1:**
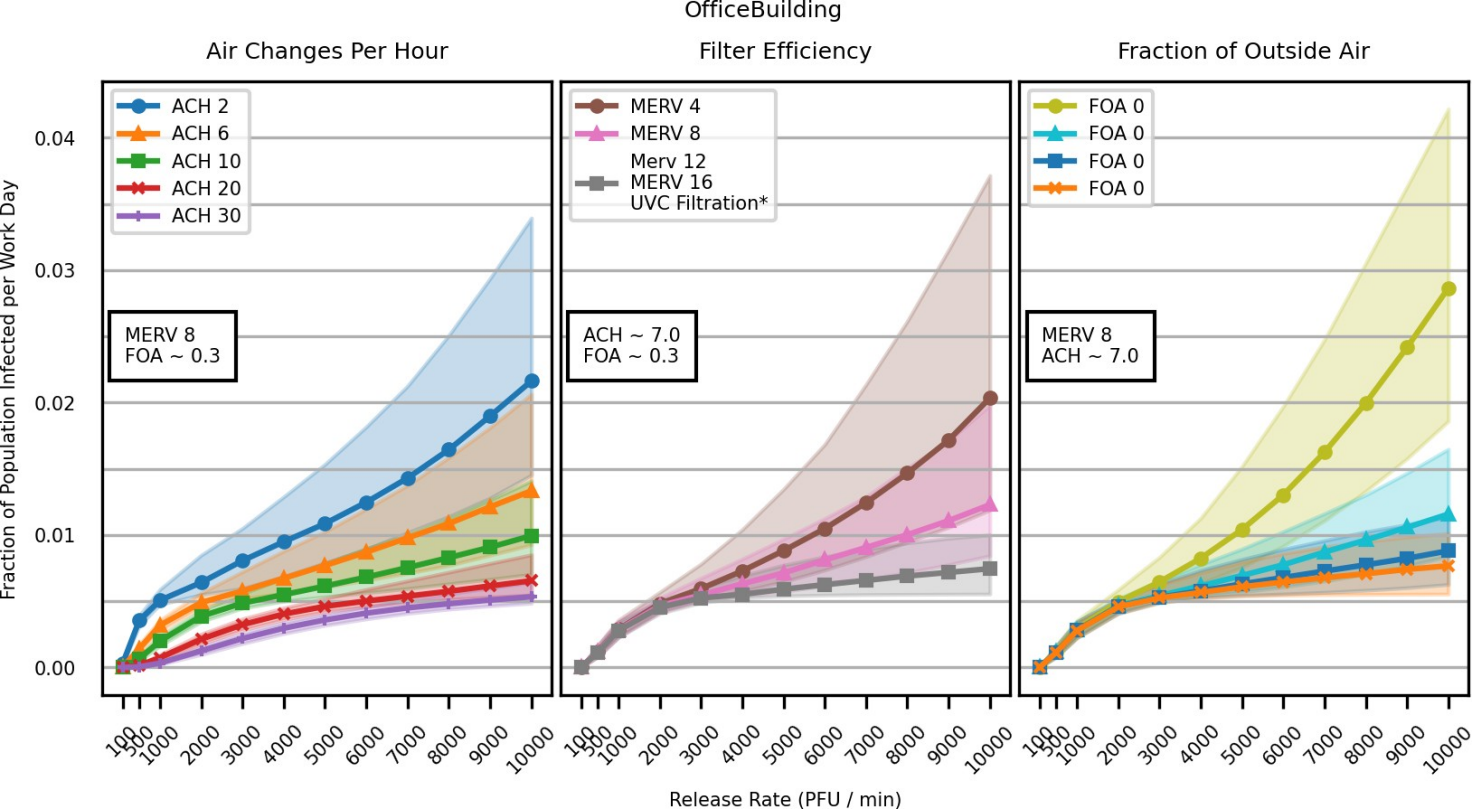
The fraction of individuals infected in simulated office buildings increases with the assumed viral emission rate but can be mitigated by improvements to common HVAC systems. Lines and points represent the median of 1,000 simulations, while shaded regions encompass the 25th to 75th percentile of results. *MERV 12, MERV 16, and any combination of UVC filtration of 90% and 99% efficiency with any mechanical (MERV-rated) filter produced nearly identical results.

All of the primary HVAC mitigation strategies impacted SARS-CoV-2 transmission in the office, quantified by the proportion of individuals in a building who became infected during a typical workday (Fig 1). The office was modeled with a population of 1082 persons, meaning a fraction of 0.01 represents approximately 10 infected persons.

In general, increasing the ACH substantially reduced transmission (Table 2), while increasing filter efficiency (MERV rating) and the FOA had smaller benefits. Additionally, ACH was the only mitigation strategy that showed efficacy at low aerosol emission rates (e.g., 100–1,000 PFU / min); the benefits of increasing the MERV rating of an HVAC filter or the fraction of outside air were not seen below approximately 3,000 PFU / min. UVC filtration was about as effective as a MERV 16 filter, regardless of the actual MERV filter used in the simulation (Fig 1).

**Table 2:** Increasing ACH, filter MERV rating, and the FOA reduce simulated SARS-CoV-2 infections with an emission rate of 3,000 PFU / min. UVC filtration and in-room air filters also reduce SARS-CoV-2 infections. Percent changes are shown relative to the highlighted baseline for each mitigation strategy (i.e., 0% change).

Building air circulation			HVAC filter rating			Fraction of outside air		
*ACH*	*Fraction Infected*	*% Change*	*MERV*	*Fraction Infected*	*% Change*	*FOA*	*Fraction Infected*	*% Change*
2	0.0081	+47.0	4	0.0060	+8.8	0.1	0.0065	+18.0
6	0.0058	+6.0	8	0.0055	0	0.299	0.0055	0
7	0.0055	0	12	0.0052	-4.1	0.3	0.0054	-1.0
10	0.0049	-11.0	16	0.0052	-4.1	0.5	0.0053	-3.3
20	0.0032	-41.0	UVC*	0.0053	-4.0	0.9	0.0053	-4.0
30	0.0022	-60.0	In-room (release)	0.0048	-11.6			
			In-room (all)	0.0047	-13.3			

*UVC filtration of 90% and 99% efficiency with any mechanical (MERV-rated) filter produced nearly identical results.

**Fig 2:**
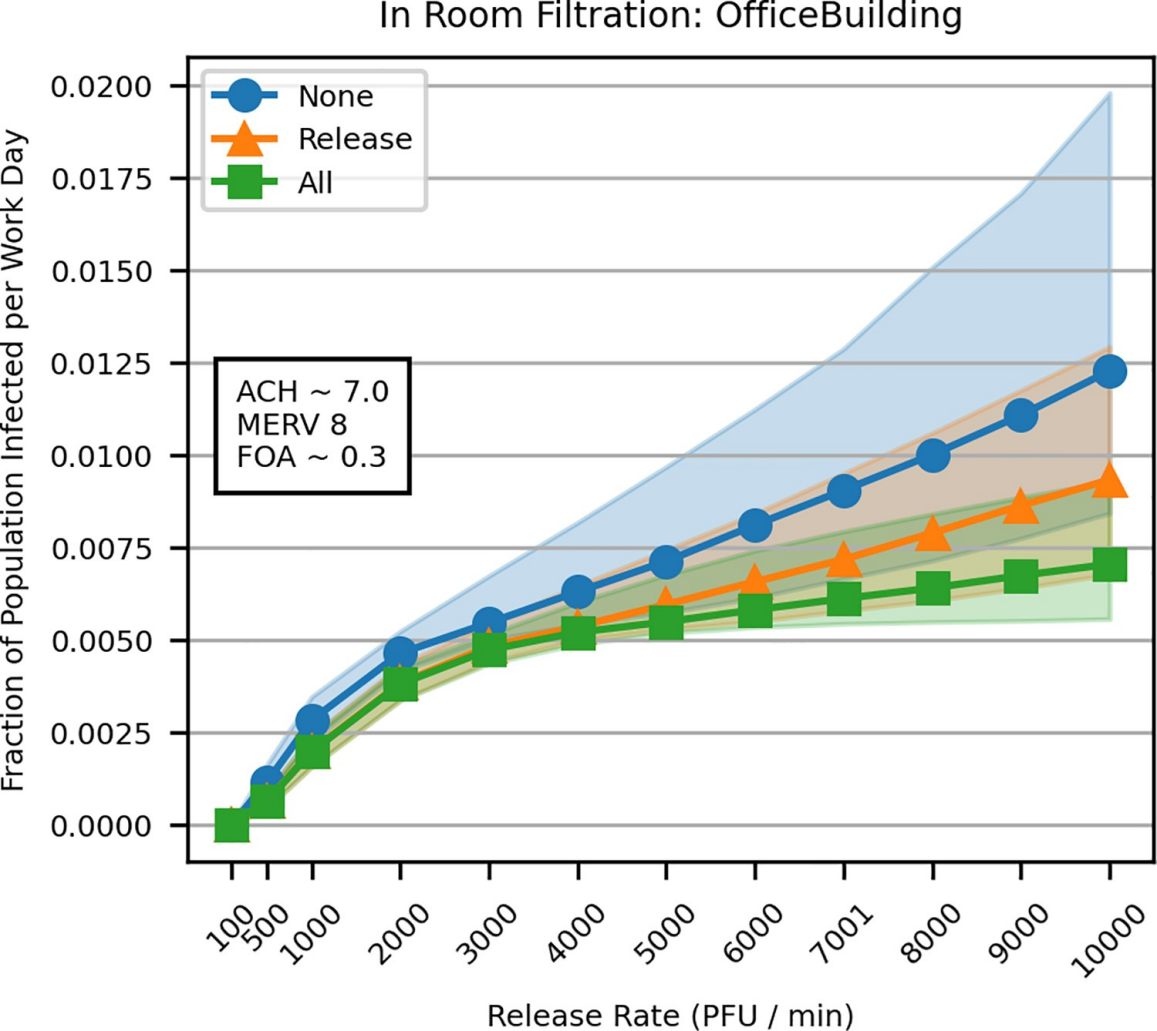
In-room filtration unit in the room of the SARS-CoV-2 release (“Release”, orange) and in all rooms of an office building (“All”, green) compared to no in-room filters (“None”, blue). Lines and points represent the median of 1,000 simulations, while shaded regions encompass the 25^th^ to 75^th^ percentile of results.

Placing an in-room filtration unit in the room of the simulated SARS-CoV-2 release reduced the proportion of individuals infected during a typical workday (Fig 2 and Table 2). Adding in-room filtration units to all rooms in an office building reduced the median proportion infected further still, though the benefit of placing units in all rooms is only visible with emission rates higher than >4,000 PFU / min. Functionally, the in-room filtration unit served to increase the amount of filtered air in the release zone, similar to increases in ACH, which also showed benefits at low aerosol emission rates (Fig 1).

The addition of UVC filters also reduced simulated infections (Table 2). In fact, UVC efficiencies of either 90% or 99% coupled with any mechanical filter produced nearly identical results. This suggests that UVC decontamination may be effective at augmenting poorer filters. We note, however, that we did not model differences in UVC filtration efficiency due to changes in particle size or HVAC flow velocity, which may affect inactivation rates. However, even with 90% efficiency, which is more conservative than both academic [31] and commercial estimates [32], in-line UVC systems reduced SARS-CoV-2 transmission in our model.

In all modeled scenarios, individuals with offices in the release zone were the most likely to become infected, followed by those individuals who visited the release zone at some point throughout the day (we explore this in more detail in Appendix B in S1 Text).

This leads to two main findings from our results for office scenarios: HVAC systems cannot entirely eliminate risk to individuals in buildings; and the tuning of HVAC settings to achieve mitigation exhibits diminishing returns.

With very low SARS-CoV-2 emission rates (e.g., 100 PFU / min), it was possible to record no infections in a simulated office day. However, HVAC systems are unable to completely eliminate infection risk at most modeled emission rates. Even at high ACH rates (Fig 1) or in scenarios with in-room filtration units (Fig 2), simulated SARS-CoV-2 infections persist. This general phenomenon suggests that persons in the near vicinity of the release, who are most likely to be infected, are the least likely to benefit from significant HVAC improvements. That is to say, individuals who happen to be near an infectious individual emitting SARS-CoV-2 aerosol are difficult to protect with HVAC systems. This is explored in Appendix B in S1 Text.

We also found that improvements to HVAC systems exhibited diminishing returns; increasing ACH, filtration efficiency, and FOA reduces simulated SARS-CoV-2 infections, and the relative benefits decrease as settings are increased (Figs 1 and 2 and Table 2). Consider the ACH results in Table 2. Increasing ACH from 2 to 6, results in 28% fewer infections (from 0.0081% to 0.0058%), which is more impactful than increasing it from 6 to 10 (15% reduction, from 0.0058% to 0.0049%). These diminishing returns are even more pronounced with filtration efficiency, where increases in MERV rating approach a point beyond which increasing benefit is not realized. In fact, for the office scenario, model predictions using MERV 12 and MERV 16 are almost identical and provide similar levels of benefit compared to MERV 8 filters (Table 2). Similarly, increasing FOA also approaches the same minimal curve as that of increasing filtration efficiency. Though increasing the fraction of outside air from 0.1 to 0.3 is highly impactful, increasing the fraction of outside air from 0.5 to 0.9 had minimal effect on the number of infections (Table 2). Benefit from increased filtration efficiency and increased FOA reach a saturation point because air can only be filtered as fast as it is pumped through a system. In contrast, we did not see a saturation point with increases in ACH. Though predicted benefits for increasing ACH show diminishing returns, the absolute benefit is only limited by situational constraints of real-world HVAC systems, e.g. the physical limitations of the systems fans.

### 2.2 Social gathering scenario results

**Fig 3:**
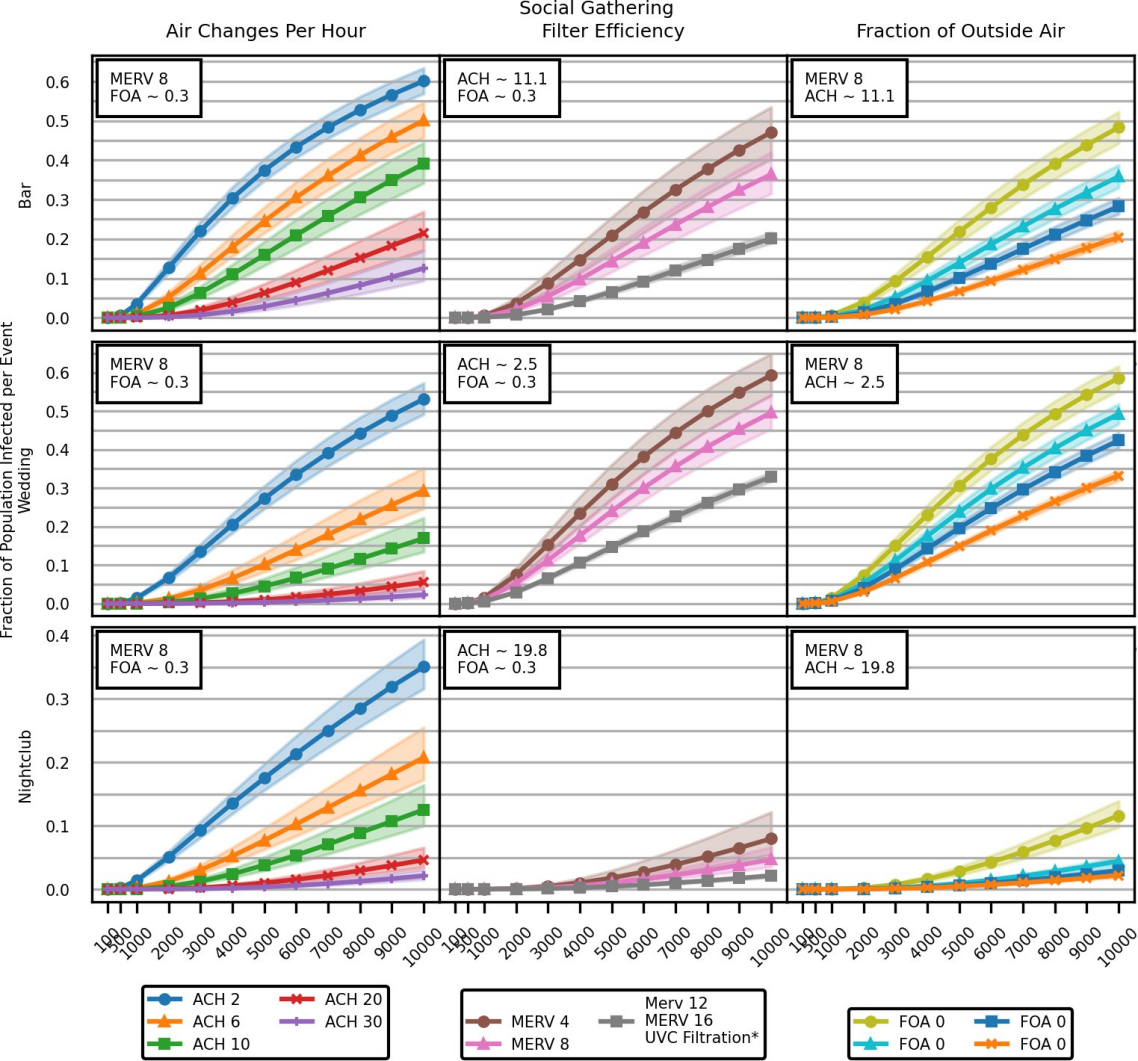
The fraction of individuals infected in simulated social gatherings increases with the assumed viral emission rate but can be mitigated by improvements to common HVAC systems. Lines and points represent the median of 1,000 simulations, while shaded regions encompass the 25^th^ to 75^th^ percentile of results. *MERV 12, MERV 16, and any combination of UVC filtration of 90% and 99% efficiency with any mechanical (MERV-rated) filter produced very similar results.

Model results from social gathering scenarios generally followed the trends observed in office scenarios. Increasing ACH, filtration efficiency, and FOA from typical values results in fewer infections, and the benefit of further increasing these parameter values decreases as they are increased. Fig 3 shows absolute results for the Bar, Wedding, and Nightclub scenarios, and Table 3 provides relative changes for a set emission rate (3,000 PFU / min).

**Table 3:** Increasing ACH, filter MERV rating, and the FOA reduce simulated SARS-CoV-2 infections in the bar/restaurant, wedding reception venue, and nightclub with an emission rate of 3,000 PFU / min. UVC filtration also reduced SARS-CoV-2 infections. Percent changes are shown relative to the highlighted baseline for each mitigation strategy (i.e., 0% change).

Building air circulation			HVAC filter rating			Fraction Outside Air		
*ACH*	*Fraction Infected*	*% Change*	*MERV*	*Fraction Infected*	*% Change*	*FOA*	*Fraction Infected*	*% Change*
**Bar**								
2	0.220	+305	MERV 4	0.088	+6	0.1	0.093	+70
6	0.113	+108	MERV 8*	0.054	0	~0.299*	0.054	0
10	0.063	+16	MERV 12	0.024	-57	0.3	0.053	-4
~11*	0.054	0	MERV 16	0.021	-61	0.5	0.036	-35
20	0.019	-65	UVC**	0.021	-61	0.9	0.022	-60
30	0.007	-87						
**Wedding**								
2	0.1347	+20	MERV 4	0.153	+36	0.1	0.150	+34
~2.5*	0.112	0	MERV 8*	0.112	0	~0.299*	0.112	0
6	0.0350	-69	MERV 12	0.069	-38	0.3	0.111	-1
10	0.0125	-89	MERV 16	0.065	-42	0.5	0.089	-21
20	0.0019	-98	UVC**	0.065	-42	0.9	0.066	-41
30	0.0005	-100						
**Nightclub**								
2	0.0932	+3,763	MERV 4	0.0045	+85	0.1	0.0073	+202
6	0.0302	+1,152	MERV 8*	0.0024	0	~0.299*	0.0024	0
10	0.0124	+412	MERV 12	0.0010	-57	0.3	0.0022	-8
~19.8*	0.0024	0	MERV 16	0.0010	-59	0.5	0.0015	-40
20	0.0024	-2	UVC**	0.0010	-59	0.9	0.0010	-58
30	0.0007	-71						

*Values for typical social gathering settings used as baseline for illustration

**UVC filtration of 90% and 99% efficiency with any mechanical (MERV-rated) filter produced very similar results.

As with office scenarios, increasing air circulation rates (ACH) provided consistent reductions in infection prevalence in the social gathering scenarios, regardless of specific venue (Fig 3). However, results for the individual social event scenarios had different responses to the increasing FOA. The absolute effect of increasing the fraction of outside air was strongly influenced by the typical air changes per hour, which was modeled as very high for nightclubs, moderate for bars, and low for wedding receptions. When the baseline ACH was high (e.g., nightclubs), increasing FOA had less of an effect on the absolute fraction of infections, which were low to begin with. Wedding receptions had the highest infection rate for any value of outside air fraction. The nightclub results show that reducing the fraction of outside air (from 0.299 to 0.1) resulted in a 200% increase in infections.

For the wedding reception scenario, infection risk remained high even with MERV 12 filters or UVC light decontamination, primarily due to the low baseline air recirculation rate in the scenario of ~2.5 air changes per hour. Functionally, only as much air as is pushed through the HVAC system can be cleaned by filters or replaced with outside air. As can be seen in Fig 3, at higher levels of air changes per hour, the wedding reception scenario predicts far fewer infections than with the expected air change rate.

**Fig 4:**
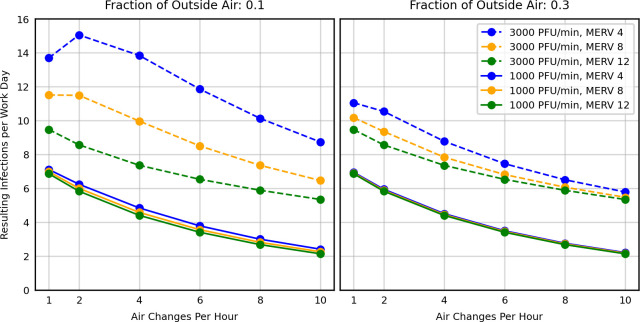
HVAC systems can facilitate spread in very limited settings, and to a relatively small extent. When air circulation rates are low and most air is being recirculated instead of pulled from outside, increasing the air exchange rate can increase the number of individuals infected outside the immediate release zone.

One important feature of our results is the relative difference in fraction infected in the office vs. social gathering scenarios. The maximum fraction infected across the social gathering scenarios ranges from ~35–60% (Fig 4), whereas fewer than 2% of individuals were typically infected in the office building scenarios (Fig 1). Office buildings contained multiple rooms on multiple floors, whereas our social gathering spaces consist primarily of single large rooms. This room alignment leads to a lower total fraction of the population infected in office building releases, though the fraction of infected individuals in the release zone of an office building can still be high (Appendix B in S1 Text).

### 2.3 Are there any cases where improving HVAC settings increased infections?

In all scenarios shown in Figs 1 and 3, simulated SARS-CoV-2 infections decreased with increasing mitigation measure, e.g., ACH = 2 to ACH = 8. That is, we initially found no cases where HVAC systems facilitated spread in an office setting. To further explore the possibility of HVAC systems increasing SARS-CoV-2 transmission, we expanded our parameter range, performing additional office model runs by systematically selecting all permutations of the parameter values listed in Table 4.

**Table 4:** Parameter values covering a wide range of possible HVAC configurations.

Parameter	Values
**Release Rate (PFU / min)**	100, 1000, 3000
**ACH**	1, 2, 4, 6, 8, 10
**FOA**	0.1, 0.3, 0.5
**Filter MERV Rating**	4, 8, 12

The results in Fig 4 corroborate the hypothesis that poor ventilation can contribute to infectious spread when ACH, FOA, and filtration efficiency are all low and the emission rate is high [5]. Specifically, model predictions suggest that an increase in ACH can facilitate spread when an HVAC system meets *all* of the following conditions:

- Low air flow: ACH less than 2
- Low FOA: FOA of approximately 0.1
- Medium to low efficiency filters: MERV 8 and lower
- High viral emission levels: greater than 1,000 PFU / min

In these scenarios, increasing ACH resulted in slightly more infections outside the immediate release zone. Because HVAC systems meeting ASHRAE minimum quality standards do not meet all of these criteria, these model predictions indicate that standard-quality HVAC systems are unlikely to contribute to or facilitate SARS-CoV-2 infections.

### 2.4 Model validation

To determine whether our model produced results in line with real-world data, we approximated the building setup explored by Hwang et al. [4], representing a multi-floor apartment complex. In this real-world setting, an infectious individual living in one apartment is believed to have infected 6 others on separate floors connected by a shared ventilation duct (“Line A” in Hwang et al. [4]). We simulated 13 apartments connected by a single ventilation duct, with an average of 1.6 people per apartment (though only a single individual in the source apartment, for 20 total individuals, one of which was the SARS-CoV-2 emission source). Because the emission rate of SARS-CoV-2 was not known in this scenario, we simulated a range of emission rates (100–3,000 PFU/min) and estimated the fraction of individuals in shared apartments that became infected after three days of emission from the source individual. A major limitation of this validation is the unknown air transfer rates between apartments through the natural ventilation shaft. To capture the uncertainty, we sampled from a widened range of indoor air speeds which governs the airflow between zones in our model: 0.05–0.3 *m/s*.

**Fig 5:**
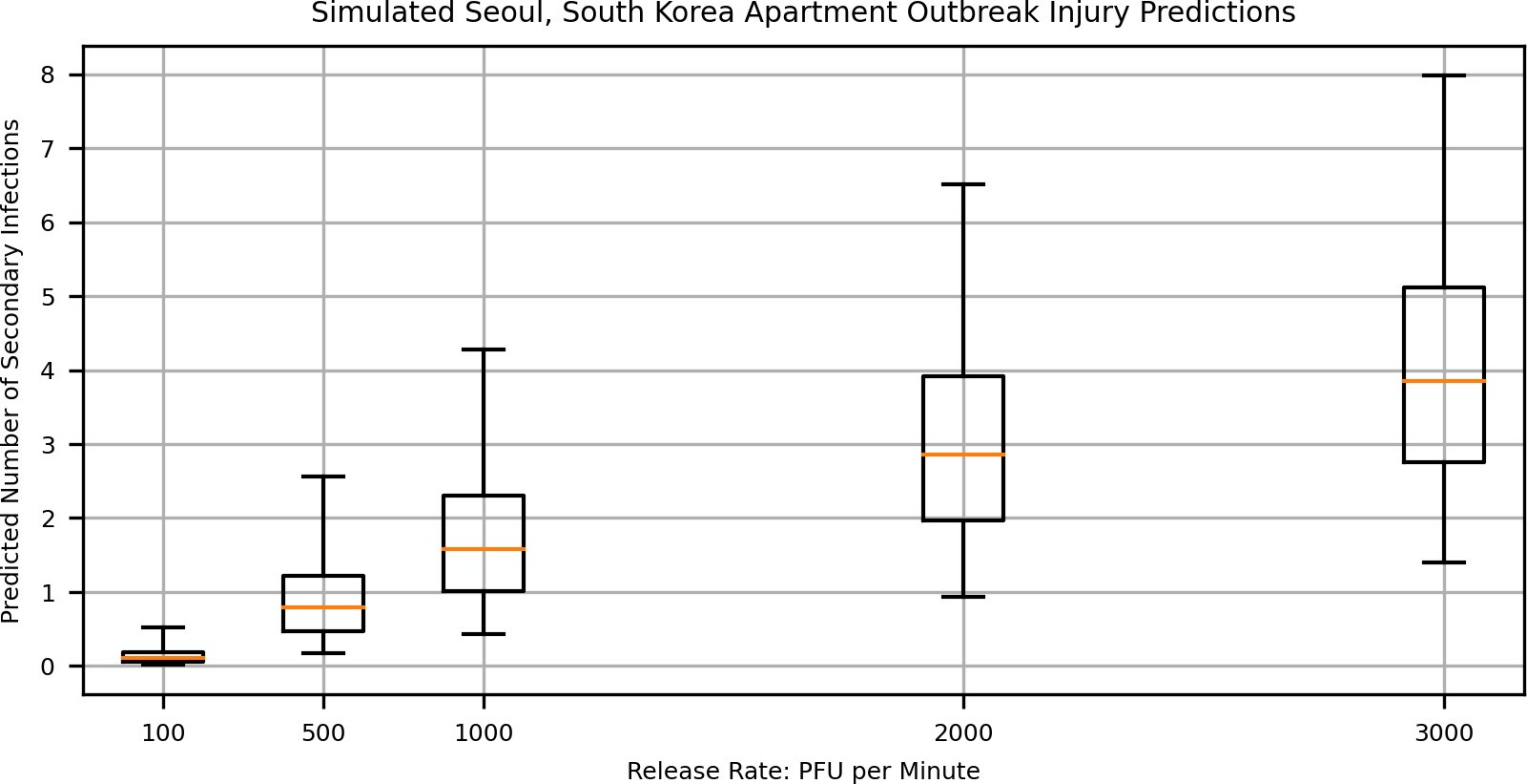
Predicted number of infections resulting from a simulated three-day release in apartments connected in a multistory complex. Horizontal lines indicate the median of 1,000 stochastic realizations, boxes represent the interquartile range, and whiskers extend to the minimum and maximum of all realizations.

The median number of infections in our simulations varied from 0.1 to 3.8, depending on the assumed emission rate (Fig 5). This is lower than the actual number of infections reported in Hwang et al. [4] (7 out of approximately 19 in “Line A”, though the number of individuals living in connected apartments was not actually given), except at the highest emission rates (3,000 PFU/min), where the maximum number of infected individuals was 8.0. Because our model is unable to simulate “secondary” transmission events (i.e. it is impossible for someone infected on day one in our model to subsequently infect someone else on a later day), and we only simulate infections resulting from the initial source, a lower number of infections is expected. In the apartment scenario, infections took place over approximately nine days. This is enough time for individuals infected early in the outbreak to infect others, given the short incubation time of COVID-19 infection [33] and high infectivity around or even before symptom onset [34].

Overall, then, our model predicts reasonable possibilities for secondary infections under the conditions investigated (Fig 5), demonstrating some real-world plausibility despite a less-than-ideal validation case. Only for the release rate of 100 PFU/min are the predictions low enough to make the apartment scenario unlikely, and even then, the median prediction suggests that there is approximately a 10% chance of 1 out of 19 persons in connected apartments becoming infected. Additionally, we did not find high rates of infection, which would suggest that our modeling approach overestimates indoor infection risk. Interestingly, the validation revealed that under circumstances of extended exposure, and for HVAC systems which perform closer to residential standards for fresh air rates and filtration efficiencies [35], HVAC performance may play a more significant role in the possibility of infection for people living in proximity to others who are infected. However, these possibilities were not investigated further.

### 2.5 Comparison with Buonanno et al., 2020

Other studies done by Buonanno et al. [21], Bazant and Bush [22], and Miller et al. [23] have used a similar approach to the Wells-Riley model in studying infection probability of SARS-CoV-2, approximating aerosols in indoor spaces as being well-mixed and reduced in concentration over time by ventilation, settling, and biological inactivation. Here we compare the approach of Buonanno et al. [21] to ours, using a similar restaurant scenario to examine differences in results. A comparison of key parameters used in the two studies, with approximate conversions where units differed, is given in Table 5. Note these are a subset of the parameter values used in this work.

**Table 5:** Parameter comparisons between Buonanno et al. and this study for a representative restaurant exposure scenario.

Parameter	Buonanno et al. value	Our value	Notes
**Ventilation**	0.5, 2.2*	0.6–2.1^*†*^	*Natural ventilation (lower value) and mechanical ventilation (higher value) ^*†*^ Ventilation without filtration is the product of ACH and FOA
**ACH**	NA	2–7	
**FOA**	NA	0.3	
**Mechanical filtration efficiency (MERV)**	0%	70–85%	Mechanical filtration efficiency in our study based on MERV 8 and particle size (MMAD) of 4.0 *μm* with GSD = 2
**Particle deposition rate**	0.004 per min [36]*	0.01 per min^*†*^	*From measurements on settled dust ^*†*^From Stokes’ Law for particle settling
**Infectious dose conversion (ID**_**50**_ **to quanta)**	1 quanta ≅ 469 PFU* 240 PFU ≅ 0.69 quanta^*†*^	240 PFU (ID_50_)	*Based on PFU necessary to produce 63% chance of infection which defines 1 quanta ^*†*^Based on quanta necessary to produce 50% chance of infection which defines the ID_50_
**Emission rate**	2.37 quanta / min ≅ 819–1,110 PFU / min*	1,000 PFU / min	
**Restaurant residence time**	90 minutes	45–90 minutes*	*Stochastic draw
**Restaurant population**	84	10–70 individuals*	*Stochastic draw
**Restaurant volume**	300 m^3^	~400 m^3^	

Buonanno et al. estimated the emission rate from an asymptomatic individual, using data on SARS-CoV-2 concentrations in sputum and considering different particle sizes emitted while breathing, whispering, and talking [21]. They used their derived emission rate values to estimate infection risk for multiple public indoor spaces using a single-zone model with well-mixed aerosol concentrations. Similar to our approach, Buonanno et al. applied ventilation (but not mechanical filtration), deposition (settling), and biological decay to the aerosol concentration of SARS-CoV-2 (21). Ventilation rates in Buonanno et al. were either “natural” without mechanical assistance, or “mechanical” with circulation by an HVAC system [21]. This definition of ventilation combines air exchange rates (ACH) with the fraction of outside air (FOA) and is comparable to multiplying the two values together in our study. Their value of 2.2 for mechanical ventilation compares well with the result of multiplying the expected values of ACH (~7) and FOA (~0.3) for our office building scenario of 2.1. The natural ventilation rate of 0.5 compares best with scenarios in our study employing an ACH of 2, which results in a ventilation rate of ~0.6 when paired with an FOA of ~0.3.

**Fig 6:**
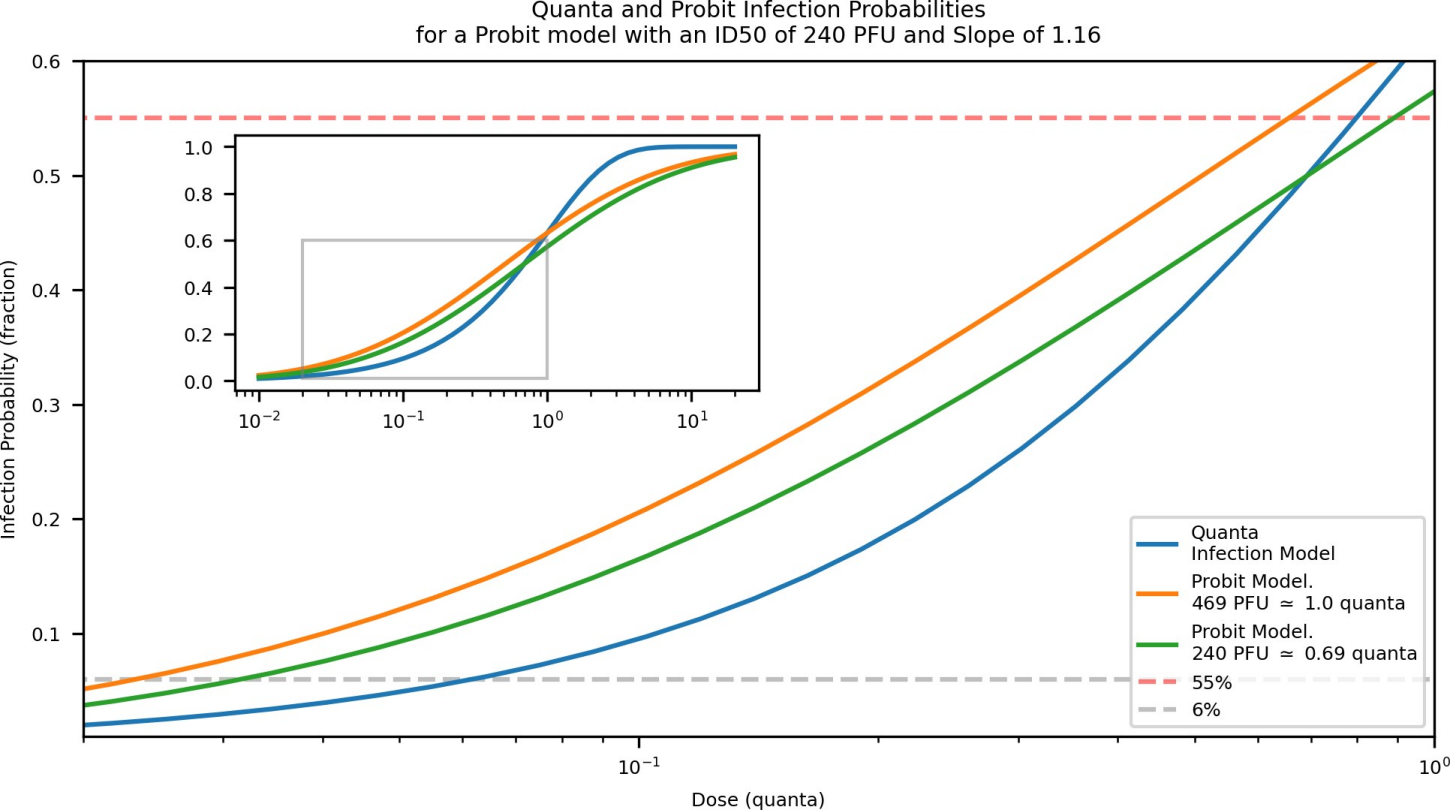
Infection probability as a function of dose for the Wells-Riley quanta of infection model and a Probit model with an ID_50_ of 240 PFU and Slope of 1.16. Two translations between quanta and PFU are shown, one using the PFU required to achieve an infection probability of 63% which defines one quanta (1 *quanta* ≅ 469 *PFU*) and the other using the quanta required to achieve an infection probability of 50% which defines the ID50 (240 *PFU* ≅ 0.69 *quanta*).

In keeping with the Wells-Riley approach, Buonanno et al. employed the “quanta” of infection, where a quanta is the dose associated with a 63% chance of infection [21]. This contrasts with our approach which used a probit dose-response model [25]. For their investigations, Buonanno et al. estimated a release rate of 142 quanta per hour (2.37 quanta per min) to be representative of a worst-case scenario for an asymptomatic spreader [21]. The quanta-of-infection model and probit models are both non-linear and there is no direct translation between them. An estimate for the range of values, which could be used to translate between the two models, can be determined from their definitions. Using the quanta required to achieve an infectious dose of 50% with an ID_50_ of 240 PFU, we get 240 *PFU* ≅ 0.69 *quanta*. Using the PFU required to achieve a 63% chance of infection (which defines 1 quanta) for a probit model with ID_50_ of 240 and slope of 1.16, we get 1 *quanta* ≅ 469 *PFU*. Fig 6 shows the resulting infection probabilities from comparative doses using a quanta-based and probit-based models.

Using these conversions, we estimate their quanta release rate to be between 819 and 1,110 PFU / min. For their restaurant scenario, Buonanno et al. predicted ~47 infections or ~55% of attendees resulting from one spreader for the natural ventilation case, and ~5 infections or ~6% of attendees resulting from the mechanical ventilation case [21]. In contrast, our model predicts fewer than 5% of infected patrons in the bar/restaurant scenario which is most comparable to theirs (1,000 PFU/min release rate, ACH = 2). With higher ACH, our model resulted in infections in fewer than 1% of patrons (Fig 3). Overall, then, our model predicted fewer infections than the model of Buonanno et al. for a roughly comparable restaurant scenario [21].

## 3.0 Discussion

### 3.1 Findings

Overall, our results show that SARS-CoV-2 transmission via aerosol is unlikely to be facilitated by HVAC systems that perform according to commercial standards, and that several common mitigation strategies are effective at reducing infection risk in different buildings. Specifically, increasing air circulation (ACH), mechanical filter efficiency (MERV rating), and FOA reduced simulated disease transmission. Additionally, the presence of UVC filters (in addition to mechanical pre-filters) functionally improved the MERV rating of less efficient filters, and the use of in-room filtration units was able to reduce transmission without corresponding increases in ACH. However, these protective effects were seen most often at high viral emission rates, which may be unlikely in the absence of super-emitters [37] or multiple infectious individuals undertaking high-emission activities (e.g., singing, coughing). Additionally, our simulated HVAC modifications were unable to completely eliminate disease risk, suggesting that individuals near a release zone or infected individual are unlikely to be protected from infection, even with high-efficiency HVAC systems.

In limited circumstances, increasing ACH in the office scenarios resulted in a small increase in expected infections during a typical day. Specifically, when there was a high viral release rate (>1,000 PFU / min) in a building with poor ventilation (<2 ACH and 0.1 FOA) and poor filtration (MERV 4), there was a slight increase in expected infections when the ACH is increased by a small amount (e.g., from 1 to 2). This occurred because the enhanced circulation slightly elevates the likelihood of individuals in neighboring rooms acquiring an infectious dose, and in this narrow parameter range, the fraction of outside air and mechanical filtration are unable to sufficiently remove the infectious particles that were spread by the enhanced airflow. This is similar, but not identical to the purported facilitation of transmission in a restaurant in China [5], where directional flow from a wall-mounted air-conditioning unit was associated with aerosol spread of SARS-CoV-2. However, the simulated office building in our scenario would not meet minimum ASHRAE air quality standards [38], making such an effect unlikely in typical settings. Additionally, increasing ACH to 4 and above reduced simulated SARS-CoV-2 infections, demonstrating the limited parameter range to which this facilitatory effect is restrained.

Interestingly, our model validation simulations suggested that infection risk in residential locations may be more sensitive to changes in HVAC settings, as typical residential HVAC standards are lower than commercial ones. Because residential systems generally introduce less outside air and use lower-efficiency filters, improvements in those features may produce a relatively large reduction in indoor infection risk. This is consistent with results for commercial settings, where the relative impact of feature changes like ACH and FOA was found to be highest with low-performance systems, with smaller relative increases as HVAC system performance increased (e.g., Table 2). Validation results also suggested that HVAC systems may play a larger role when exposure periods are longer than those experienced in a typical workday or social event, though we did not explore this in detail in this study.

In general, increasing ACH was the most effective protective measure, as increased airflow produced large reductions in infection prevalence in both office and social gathering environments (Tables 2 and 3). This was particularly true in the wedding scenarios, where a low ACH was assumed by default. Importantly, increasing ACH reduced simulated infections even at low aerosol emission rates (e.g., <1,000 PFU / min), and this effect was visible in both the office and social gathering environments. Increasing ACH provides more benefits in the room of release than increasing FOA or MERV, primarily because it can directly reduce aerosol SARS-CoV-2 concentrations. Realistically, however, increasing ACH is not cost-effective in all situations, in terms of reduction in infection risk relative to any increase in energy costs. In hot or humid environments, for instance, the additional energy expenditure associated with conditioning outside air may be greater than the extra energy costs associated with increasing mechanical filtration efficiency, making reductions in infection risk via an increase in filter MERV rating more feasible than changing ACH or FOA [39]. While we did not model this specifically, it is likely that increasing ACH, FOA, and filter MERV ratings have synergistic effects; increasing ACH increases the rate at which in-room air gets filtered, for instance, enhancing the benefits from more efficient filters. Overall, it is important to consider the real-world context of a given building when designing effective strategies for reducing indoor infection risk via HVAC systems [39].

In no instances were HVAC systems able to fully reduce infection risk, primarily because HVAC systems could not totally mitigate risk to those individuals who occupied the same room or zone as an infected individual. Increasing ACH or adding in-room filtration units, however, did show some ability to reduce in-zone infections (e.g., single zone nightclubs benefitted tremendously from higher ACH) primarily at higher emission rates (e.g., >1,000 PFU / min). In the office building, most infections occurred in individuals who “worked” in the release zone or in individuals who visited the release zone at some point during the day; very few individuals without this direct exposure became infected (Appendix B in S1 Text). For this reason, all of our primary mitigation measures (ACH, MERV, FOA) exhibited diminishing returns. Beyond a certain point, virtually all risk to those outside of the release zone is minimized, but our simulated HVAC systems could not entirely eliminate risk to those closest to the source of infection. We only saw scenarios with zero predicted infections when emission rates were low, e.g. <1,000 PFU/min.

When comparing our results with a roughly similar restaurant scenario modeled in Buonanno et al., 2020, we found fewer infections at two different ventilation rates [21]. The difference in results is likely influenced most heavily by the presence of the mechanical filter in our model, which was absent in theirs. For the comparison, we used a MERV 8 filter, standard for commercial spaces in the United States, which are rated with an efficiency between 70% and 85% for particles between 3.0 and 10.0 μm in diameter (capturing our aerosol particle MMAD of 4 μm). When paired with an ACH of 2, as in their natural ventilation scenario, this efficiency represents an additional decay of ~2.6% per minute, roughly triple the natural ventilation decay of ~0.83% per minute. Even with low ACH, then, our addition of mechanical filtration eliminates significantly more aerosol particles from circulation, helping to explain the lower infection rates estimated by our model. Additionally, Buonanno et al. had more people in their restaurant scenario, for a longer period of time, with a smaller air volume, with a lower particle settling rate (Table 5), all of which would tend to increase infection prevalence relative to our study [21]. Functional differences in the quanta and probit dose-response curves (Fig 6) also complicate direct comparisons.

### 3.2 Limitations

Our model and analysis are subject to several limitations. Estimates of aerosol emission rates for SARS-CoV-2 remain a key gap in current understanding. We simulated a range of emission rates over two orders of magnitude (100–10,000 PFU / min) to account for uncertainty in this key parameter, but a more realistic estimate would help refine our results to allow better comparisons to other studies. For instance, United States Transportation Command (USTRANSCOM) conducted an aerosol tracer study inside commercial aircraft to estimate infection risk on flights. Their experiments simulated a single point source release, like our model, with a release rate of 4,000 PFU / hour [40]. This equates to 66.7 PFU / min, which is less than our lowest aerosol parameter. The study identified a low risk of aerosol infection on flights due to high air circulation rates [40], though our results (e.g., Fig 1) suggest this may be as much a function of the low emission rate or the high infectious dose they assumed (1,000 PFU) as the protective effect of an air handling system. While our simulated range is in line with real-world data (Table 6), those estimates require several conversion factors, and a better understanding of emission rates would improve consistency across studies.

**Table 6:** Estimated SARS-CoV-2 aerosol emission rates via exhaled breath for a single individual.

RNA copies per hour [52]	RNA copies per minute	RNA:PFU ratio (8)	PFU / min
**22,500,000.00**	375,000.00	25.00	15,000.00
**22,500,000.00**	375,000.00	163.63	2,291.76
**22,500,000.00**	375,000.00	200.00	1,875.00
**22,500,000.00**	375,000.00	1,666.70	225.00
**22,500,000.00**	375,000.00	2,000.00	187.50
**103,000.00**	1,716.67	25.00	68.67
**103,000.00**	1,716.67	163.63	10.49
**103,000.00**	1,716.67	200.00	8.58
**103,000.00**	1,716.67	1,666.70	1.03
**103,000.00**	1,716.67	2,000.00	0.86

Limitations also exists in the particle distribution used to model the SARS-CoV-2 aerosols. Modern experimentation has shown exhaled aerosol distributions to be multi-modal and vary with type of respiratory activity [41]. We have modeled it here using a unimodal, log-normal distribution, and discuss implications of this single distribution in Section 4.2.3. Additionally, deposition of inhaled aerosols in the respiratory track has been shown to be dependent on particle size [42,43] where we have modeled all particles between 1 and 10 microns as contributing to dose. While we model larger particles as containing more virus (in units of PFU), which increases their probability to infect compared to smaller particles on a per-particle basis, we do not consider the location of deposition in the respiratory tract as a contributing factor to infectious dose or pathogen virulence.

We did not consider the effect of individual face masks on either the “infected” individual or others inside our buildings. As face masks reduce both emitted and inhaled particles [44], their use would alter our results. Also, because we assumed that each building zone was well-mixed, the effect of directional breathing encountered in a close face-to-face conversation is not accounted for. Higher-resolution models may consider less restrictive assumptions about particle and air mixing within zones. Our primary results only reflect the risk from a single event or day of work. Compound effects of continued daily exposure due to an infected individual returning to work on consecutive days or repeatedly attending social events were not addressed, though the exposure length for the validation study in the residential apartment setting was extended to three days. Additionally, we considered a single point source aerosol release, and did not model multiple emitters or an infected individual moving throughout buildings during occupancy.

## 4.0 Parameters and methods

### 4.1 HVAC Parameters Investigated

We modified three primary attributes of HVAC systems and assessed their impact on disease transmission, but did not assess the feasibility of these changes in real systems:

- ACH–the frequency that a volume of air equal to the volume of air contained in the building will have been recirculated through the HVAC system. Escombe et al., linked an increase in ACH in a hospital setting with reductions in tuberculosis risk to patients [45]. Rates of 4 to 10 ACH are typical for office spaces, and ACH rates between 2 and 15 may be found in many public buildings, with rates up to 30 ACH in some public spaces [46].
- FOA–the fraction of recirculated air that comes from outside the building (i.e. “fresh air”). Luongo et al. found that a low FOA contributed to either disease prevalence or surrogates of disease prevalence such as absenteeism [47], and respiratory infections in shared military barracks decline when access to open windows is enhanced [48]. An outside air fraction of 0.1 is low for most buildings and may be considered a reasonable lower bound. Even if outside air is not purposefully introduced, it may account for other building envelope leakage. High values like 0.9 may be unachievable given climate and HVAC system specifics. Default FOA values for all scenarios were modeled using a truncated beta distribution with min of 0.04, max of 1.0, and mode of 0.25 (alpha and beta parameters of 3.26 and 7.80 respectively). The mode was picked to roughly estimate values expected for systems using outdoor air rates and equations from ASHRAE Standard 62 [38] with expected total recirculation (ACH) rates provided by Bell [46]. Because no real system can be perfectly airtight, the lower bound was increased from 0 to 0.04. A beta distribution was chosen as it allows for more probability for higher values of FOA which can account for extra building leakage beyond the design of the HVAC system.
- Filtration Efficiency–the particle-size-dependent efficiency of the mechanical filters in the HVAC system at removing contaminants from the air. The filtration efficiency values in the model use the MERV ratings developed by ASHRAE [49]. Mechanical filter ratings of MERV 4 through MERV 16 are rated by ASHRAE for specific filtration efficiencies at different particle sizes. MERV 13 filters are rated for the removal of individual virus particles from the air [13], while most household and business filters have a MERV rating of 5–8 [14].

In addition to the three primary features listed above, we simulated two alternative methods of filtration as potential mitigation strategies. These alternative methods have been postulated as potential methods to reduce COVID-19 risk and may be implemented either on their own or in tandem with other modifications to HVAC systems. These methods are:

- UVC Filtration Step–adding an ultraviolet lamp into the HVAC system after the mechanical filter. This lamp emits ultraviolet light to a small cross-section of the ducts that will inactivate some fraction of SARS-CoV-2. In an experimental setup, Walker and Ko found that UVC radiation (254 nm) reduced the amount of aerosolized murine hepatitis virus (MHV, a mouse coronavirus) by 88% with a dose of 0.66 mJ/cm^2^ [50]. In another experiment, Buonanno et al. used 222 nm UVC to inactivate >99% of two aerosolized human coronaviruses (229-E and OC43) with doses of 1.2–1.7 mJ/cm^2^ [31]. Commercially available UVC systems for HVAC use are able to produce UV doses of 5.1 mJ/cm^2^, enough to inactivate >99% of aerosolized viruses in experimental testing [32]. From these estimates, we simulated UVC filtration efficiencies of 90% and 99% in our modeling study. A brief literature review of UVC decontamination in aerosolized viruses is available in Appendix A in S1 Text.
- In-Room–adding a filtration unit (Honeywell True Allergen Remover [51]) to the room of the infected person (i.e., the release zone), or all rooms of the office. Documentation on the unit specifies an ACH of 5 for a room of area 465 *ft*^2^ (43 *m*^2^) [51]. Assuming a room height between 9 and 10 feet yields an approximate filter flow rate of 353 *ft*^3^/*min* (10 *m*^3^/*min*). The stated filtration efficiency for the unit is 99.97% for particles down to three microns in diameter [51], which was conservatively approximated as 99% efficiency given variation in modeled particle sizes.

### 4.2 SARS-CoV-2 Parameters

#### 4.2.1 Release rates for infected individuals

There is substantial uncertainty in the emission rate of SARS-CoV-2 by infectious individuals, as the relationship between individual particle emission rates, SARS-CoV-2 viral load, and transmission risk is unclear. Stadnytskyi et al. estimated that human speech produces ~1,000 virion-containing droplet nuclei per minute [3], though a single virion is not the same as a Plaque-forming Unit (PFU). Assuming variable ratios of 1 virion per PFU to 100 virions per PFU, a plausible low-end estimate is 100 PFU / min, which aligns with prior emission rate estimates for quiet speech [37]. Ma et al. used cycle threshold values from quantitative COVID-19 diagnostic tests to estimate that individuals emit between 1.03 x 10^5^ to 2.25 x 10^7^ viral genome (RNA) copies per hour in exhaled breath alone [52]. Based on the work of Fears et al., who quantified SARS-CoV-2 persistence in aerosol samples, a plausible range of RNA copies per PFU is ~25–2,000 (from their Fig 2A and 2B) [8]. From these approximate emission rate ranges and RNA:PFU ratios, we obtain crude but plausible estimates of a single individual emitting between ~1 and 15,000 PFU / min via exhaled breath (Table 6). To capture the uncertainty in individual emission rates from breathing, talking, or sneezing/coughing, we assumed a single point emission source ranging from 100 to 10,000 PFU / min.

Additional work to clarify the SARS-CoV-2 emission rate would greatly benefit this and other modeling studies, as we know that the total number of aerosol particles released by an individual depends on several factors. For instance, Morawska et al. documented differences in emission rate by vocalization type [41], Asadi et al. found substantial variation among individuals in aerosol emission rates [37], and Lindsley et al. reported that the number of aerosols produced by a cough depended on whether an individual was sick with influenza or had already recovered [53]. While these types of differences can be integrated into estimates of individual SARS-CoV-2 emission rates (as in Buonanno et al. [21]), our wide range of assumed values enables a broad understanding of how indoor infection risk varies with emission rate.

#### 4.2.2 Infectious dose

The median infectious dose (ID_50_, the amount of virus inhaled that would cause 50% of humans to become infected) of SARS-CoV-2 is also unknown. We used a prior estimate of 240 PFU with a probit slope of 1.16 from previous studies on SARS-CoV-1. This estimate uses mouse data for SARS-CoV-1, combining A/J mice infected with murine hepatitis virus, as well as transgenic hACE2 mice that express the human angiotensin-converting envzyme-2 receptor that the coronavirus uses to gain cellular entry infected with a SARS-CoV-1 knockout strain [29,30]. Older work with seasonal, non-SARS-CoV-2 coronaviruses reported lower infectious doses, on the order of tens of PFU [54–56]. Experimental identification of the human median infectious dose of SARS-CoV-2 via different exposure routes remains a data gap.

#### 4.2.3 Aerosolized particle size

Due to methodological differences as well as the effects of evaporation, there is substantial variation in the literature regarding the particle size distributions arising from talking, coughing, and sneezing. Additionally, a wide range of particle sizes (0.5–500 μm) have been shown to carry pathogens [57]. From a review by Nicas et al. [57] and studies estimating exhaled particle sizes for speaking and coughing [58,59] and sneezing [60], we estimated exhaled particle size distributions assuming a unimodal lognormal distribution (Table 7).

**Table 7:** Plausible particle size distribution ranges for various emission types.

Activity	Lower bound GM (μm)	Lower bound GSD	Lower bound reference	Upper bound GM (μm)	Upper bound GSD	Upper bound reference
**Talking**	16.0	2.6*	[58]	88.9	1.5^*†*^	[59]
**Coughing**	14.0	2.6	[57]	114.2	1.9^*†*^	[59]
**Sneezing**	8.1	2.3	[57]	360.1	1.5	[60]

*the geometric standard deviation was not given in this study, and is instead taken from coughing estimates in a separate reference [57].

† geometric standard deviations were not given in this study, and were calculated using observed distributions of particle sizes, assuming a unimodal lognormal distribution.

Post-emittance, it is understood that exhaled droplets quickly desiccate [3,22,57]. From this data, we selected the desiccated aerosolized particle MMAD of 4.0 μm identified by Stadnytskyi et al. using a GSD of 2.0 which is consistent with the estimates presented in Table 7.

Our selection of a single unimodal value is a simplification of the literature. In recent years, a number of studies have characterized the distribution of particles emitted during different activities. Morawska et al. found that the emitted particle size distribution changed with vocalization type, for instancing reporting an increase in particles around 3.5 and 5 μm during speech compared to simply breathing (modal particle size <0.8 μm) [41]. On the other hand, Asadi et al. also measured particle size number-distribution for vocalized “ah” sounds and reported no significant difference in distribution due to loudness level or language spoken [37]. Chao et al. found small differences in geometric mean particle size for coughing (13.5 μm) and speaking (16.0 μm), though their measurements were close to the mouth and precluded evaporation [58]. Our assumption of a single particle size distribution could affect our results due to the particle-size dependent model mechanics of settling and mechanical filtering (discussed in Section 4.3), as well as the determination of which inhaled particles contribute to dose received by healthy individuals (discussed in Section 4.4). Shifting our assumed distribution towards larger particles, as in the unevaporated estimates from Chao et al. [58], would cause more particles to settle out of the air at a faster rate and increase the percent of particles filtered, reducing infection risk. Shifting the distribution towards smaller particles, as in the breathing estimates from Morawska et al. [41] or the vocalizations of Asadi et al. [37], would reduce both the effects of settling and mechanical filtering on the reduction of SARS-CoV-2 aerosols, increasing infection risk compared to the results presented here.

#### 4.2.4 Biological inactivation/decay rate

The aerosol decay rate of SARS-CoV-2 has been estimated by van Doremalen et al. [28] and Schuit et al. [27]. van Doremalen et al. aerosolized SARS-CoV-2 with a Collison nebulizer at an initial concentration of 105.25 50% tissue-culture infectious dose (TCID_50_) per milliliter and a particle size of <5 μm at 21–23°C and 40% RH. Half-lives in this study were estimated by comparing reduction in infectious viral concentration. Schuit et al. estimated SARS-CoV-2 aerosol decay rates in simulated saliva in the dark (half-life ~99.0 minutes), in sunlight typical of December (half-life ~5.73 minutes), and in sunlight typical of July (half-life ~ 2.26 minutes). The Schuit et al. decay estimates include both loss of particle viability as well as particle deposition and were conducted at a variety of temperatures and relative humidity levels. From this data (Table 8), we modeled the biological inactivation for our simulated indoor environment using the mean (0.88%) of a uniform distribution from 0.7%[27] and 1.06%[28] loss per minute.

**Table 8:** Estimated aerosol decay rates for SARS-CoV-2 suspensions or simulated droplets.

Particle generation	Particle Size (μm)	Half-life (minutes)	Half-life Range (minutes)	Decay Rate (fraction per minute)	Decay Rate Range (fraction per minute)
**Collison nebulizer**	<5	65.4	38.4 to 158.4	-0.0106	-0.0180 to -0.00438
**Two fluid nozzle**	2	99.0	NS	-0.007	NS
**Two fluid nozzle**	2	5.73	NS	-0.121	NS
**Two fluid nozzle**	2	2.26	NS	-0.306	NS

### 4.3 Aerosol transport model

The transport model uses a network of zones which communicate aerosols between them using bi-directional volume exchange. Similar to approaches used by Wells, Riley, Gammaitoni and Nucci, Noakes, and others [24,61,62], the zones are modeled as being well-mixed, with uniform aerosol concentration within a single zone. Zones in the model are used to represent aggregate collections of rooms in the building being modeled or, when the rooms are very large, subdivided sections of rooms. Connections between zones in the model are used to represent doors, large openings, and air ducts, and volume exchanges are sized appropriately. Separate zones are also used to represent the HVAC units. At each time step of the model, the amount of air which is communicated between the HVAC units and other zones depends upon the building ACH setting. The amount of air that other zones exchange between each other depends upon a randomly sampled indoor air speed and the area of the opening (such as a door) across which the air is meant to move.

The aerosol transport model uses Monte Carlo sampling to draw from a list of model parameter distributions and calculate the total exposures and infections for the modeled population in the given scenario. This process is repeated for many realizations to produce a range of estimates that represent different combinations of the input parameters. Most parameters are sampled independently unless a specific dependency is enforced in the model.

**Fig 7:**
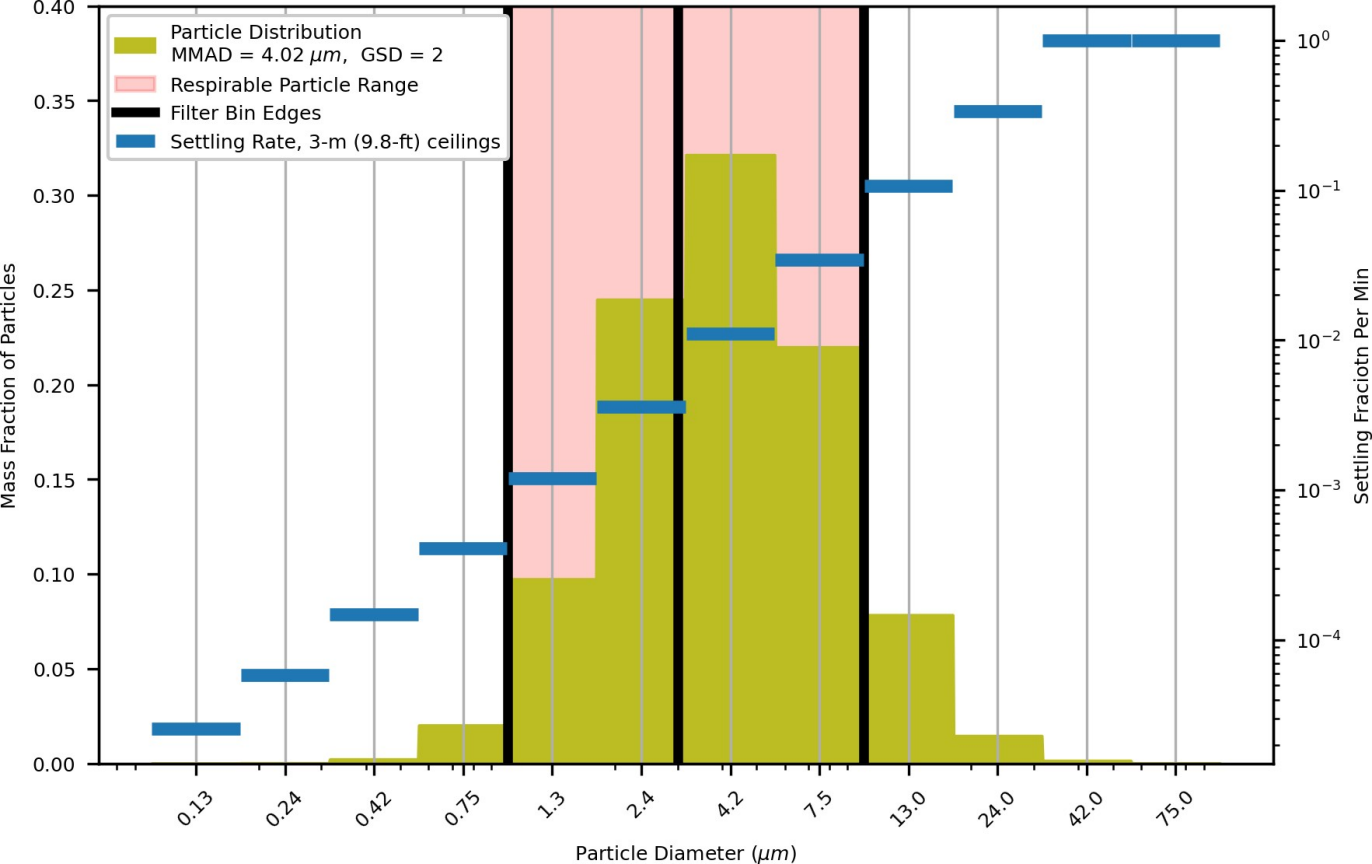
Particle distribution by mass for dry MMAD of 4.02 micrometers and a GSD of 2.0 (Section 4.3.1) with mechanical filter bins (Section 4.3.4), respirable particle range (particles that contribute to dose) (Section 4.4), and particle settling rate as calculated by Stokes’ Law (Section 4.3.2).

Aerosol generation by the infected emitter was simulated as a continuous point source in a selected building zone. Generated aerosols were distributed into 12 particle size ranges determined by a log-normal distribution using a selected MMAD of 4 μm and GSD of 2 (Section 4.2.3). Besides mixing between zones, filtration, and exchange with outside air for the air contained in the HVAC zones was also applied at each time step. Settling was applied to all aerosols, with the exception of those in an HVAC unit (HVAC zone). Filtration and settling rate were both particle-size dependent, so different filtration efficiencies and deposition rates were applied to each of the 12 bins in the aerosol particle distribution (Fig 7). Biological decay or inactivation was also applied to all aerosols (Section 4.2.4).

Where we have chosen to model the effective particle distribution as dynamic with time, applying these mechanics to modify the particles remaining in the air and thus capable of causing infection, it is also possible to estimate an effective infectious particle size *a priori*. In their recent study, for instance, Bazant and Bush [22] use similar mechanisms to identify an effective infectious droplet (aerosol particle) size, which they then use to derive a simple expression relating an allowable exposure time to the ACH, room volume, breathing rate, and virus concentration in the room aerosols.

#### 4.3.1 Contaminant generation in release zone

We model an infected individual releasing SARS-CoV-2 via exhalation into the release zone, with resulting aerosol particles resolved by both quantity (mass) and particle size. The mass released is distributed across the particle size bins according to a log-normal distribution with the mean given by the median mass aerodynamic diameter (MMAD) and a geometric standard deviation (GSD). The post-evaporation dry MMAD and GSD used for SARS-CoV-2 was 4.0 μm and 2.0 respectively (see Section 4.2.3).

#### 4.3.2 Settling of particles

Particle settling applies a loss rate using velocities calculated from a Stokes’ Law equation based on contaminant density, size of the particles, analytically derived constants, and physical properties of air [63]. Since the deposition rate depends on the size of the particles, a different deposition rate is calculated for each particle size bin.

**Equation 1**. Stokes’ Law Settling Equation for Particle Deposition

$$V_{settling}=\frac{{D_{p}}^{2}*g*(\rho_{p}-\rho_{a})*C_{c}}{18*\mu }$$

Where:

- *V*_*settling*_ is the settling velocity in meters per second
- *D*_*p*_ is the particle diameter in meters
- *g* is the acceleration due to gravity in meters per second-squared (9.8)
- *ρ*_*p*_ is the particle density in kilograms per cubic meter
- *ρ*_*a*_ is the density of air in kilograms per cubic meter (1.2041)
- *C*_*c*_ is the Cunningham correction factor
- *μ* is the air dynamic viscosity in kilograms per meter-second (1.82e-5)

The Cunningham correction factor is an adjustment to the velocity that scales with particle diameter and some experimentally derived constants. It is calculated by the following equation [64]:

**Equation 2**. Cunningham Correction Factor Equation for Particle Deposition

$$C_{c}=1+\frac{\lambda }{D_{p}}*\left(2.514+0.8*e^{\frac{-0.55*D_{p}}{\lambda }}\right)$$

Where:

- *λ* is the mean free path of air in meters (6.7e-8)
- *D*_*p*_ is the particle diameter in meters
- and 2.514, 0.8, and 0.55 are experimentally derived constants

The equation used for estimating the rate of particle deposition in each timestep is given as:

**Equation 3**. Rate of Particle Deposition Equation from Settling Velocity

$$R_{settling}=V_{settling}*\frac{60}{h_{ceiling}}$$

Where:

- *R*_*settling*_ is the rate of particle settling/deposition (1/minutes)
- *V*_*settling*_ is the settling velocity in meters per second
- *h*_*ceiling*_ is the height of the ceiling from the floor, measured in meters
- 60 is the number of seconds in a minute

The model does not consider temperature or humidity in the calculation, as these properties are not tracked in the model. As well, fomite spread of contaminant which has settled out and deposited on surfaces or the re-entrainment of those particles back into the air is not considered.

#### 4.3.3 Bi-directional mixing

For the transfer of contaminated air between zones, the model assumes equal exchange of volume across air connections. This is a simplification compared to flow models, where pressure differences and temperatures lead to air transfer which is predominantly in one direction across a given connection between rooms. This bi-directional approach is used as we desire the buildings to be generic, without pre-determined flow pathways.

The zone connections in the model are specified using a network graph. This allows any zone to exchange with any other zone if a connection is specified. Connections in the model can represent doors, large openings, or completely un-obfuscated areas between very large rooms, which have been subdivided into smaller zones. The amount of volume exchange across a connection is dictated by the area of the connection and an indoor air speed, which is drawn from a distribution for a particular model realization.

Building HVAC systems are also represented using a zone, which has connections to the areas (other zones) of the building the system services. The volume exchanged between the HVAC zone and the serviced zones is dictated by the building ACH. Air ventilated into the HVAC zones remains there for a single timestep while filtration and exchange with outdoor air are applied. This likely underestimates the time necessary for air to complete a recirculation through the HVAC system, but without specification of the size, length, and position of ductwork, calculating an accurate ventilation recirculation time is infeasible. At the following time step, air in an HVAC zone, which has been filtered, is exchanged back to the zones connected to the HVAC. Because the HVAC zones service multiple building zones, and air in the HVAC zone is considered well-mixed, the HVAC system contributes to transporting aerosols across the building.

#### 4.3.4 HVAC particle filtration and ventilation

Contaminant particles that enter the HVAC system zones will be passed through a mechanical filtration step that removes contaminant particles from the air to be recirculated. The fraction of particles filtered depends upon the particle size of the contaminant that is ventilated. For each particle size bin, a small fraction of particles was assumed to bypass the filter entirely due to pressurization of the ductwork and gaps around the filter. Other factors likely contribute to the real-world efficacy of mechanical filters, such as filter age, temperature, humidity, and air speed, though they are not considered in our model. For scenarios where UVC decontamination was simulated, it was applied as a further reduction in contaminant in the HVAC system zones during the filtration step. However, no dependency upon particle size was applied and no contaminant was assumed to bypass this type of decontamination. For a systematically selected filter MERV rating, the filtration efficiency for each particle diameter filtration bin was drawn stochastically from the efficiencies in Table 9. The 12 aerosol particle diameter distribution bins were assigned this filtration efficiency according to their log-spaced center values.

**Table 9:** MERV filter efficiency by particle diameter range for select filters.

MERV Rating	Efficiency (fraction filtered) by Particle Diameter (microns)			
	0–1	1–3	3–10	>10
4	0	0	0–0.2	0.2–1.0
8	0	0	0.7–0.85	0.85–1.0
12	0	0.8–0.9	0.9–1.0	1.0
16	0.95–1.0	0.95–1.0	0.95–1.0	1.0
UVC	0.99	0.99	0.99	0.99

Ventilation or exchange with outdoor air is also modeled as a decay term on volumes contained within the HVAC system zones using the FOA. The FOA is modeled using a beta distribution centered on a fraction of 0.25 to also allow for significant probability of higher fractions which can account for overall building leakage. Obviously, in reality, extra ventilation due to building leakage would not apply strictly to air contained in the HVAC system. Modeling it this way is a simplification.

#### 4.3.5 Biological inactivation / decay

Biological decay was applied using an exponential model with half-life data and decay fraction per minute estimates for indoor conditions (Section 4.2.4). In general, biological decay rate may be affected by environmental conditions such as temperature and humidity [27,65,66], but these effects were not modeled directly.

### 4.4 Population dose response

Indoor populations in the model were simulated using groups of people assigned simple patterns of movement between simulated zones for the duration of the simulation. At every time step, groups are assigned a respired dose according to the aerosol concentration of the zone they are located in. In the model, particles in the range between 1 and 10 are considered viable for dosing. This is a simplification of quite complicated particle-size dependent dose mechanics and is a limitation of the model. In general, the deposition of particles in the respiratory tract has much greater dependence on particle size than we model and is also dependent on location in the respiratory system (e.g., in the trachea or alveoli). This can also impact resulting infection. Focusing on the 1 to 10 micron range effectively focuses on particles that can make it to the lower respiratory tract, which is generally more susceptible to infection, though infection in the upper respiratory track or even gastrointestinal tract is possible [42,43].

#### 4.4.1 Infection probability calculations

At the end of the simulation, the probability of becoming infected with SARS-CoV-2 was calculated using a probit dose-response model. The response curve is characterized by a median infectious dose (ID_50_) and a probit slope. The ID_50_ is defined as the dose in plaque-forming units (PFU) that would represent a 50% probability of infection.

The probability of infection is calculated from the total exposure dose, dose-response curve parameters, and the standard normal distribution as given by the following equation:

**Equation 4**. Probability of Infection given Exposure Mass

$$P_{infection}=\frac{1}{2}*\left[1+erf(\frac{s*\log_{10}\left(\frac{Dose}{{ID}_{50}}\right)}{\sqrt{2}})\right]$$

Where:

- *s* is the probit slope parameter of the dose-response curve
- *Dose* is the total exposure
- *ID*_*50*_ is the median infectious dose

As all individuals within a modeled group receive the same exposure to SARS-CoV-2, the expected number of infections for a group is the probability of infection for the group multiplied by its size. The total expected number of infections for a model realization is simply the sum of expected infections across all groups.

#### 4.4.2 Population movement

Following the calculation of exposure, groups are moved to different locations. Each building model in the simulation has different population dynamics that impact the results of the model, but the basic elements of population movement are similar for all models. Each group is assigned a schedule dictating which zones they should visit and how long they should stay there. Groups may be assigned to enter or exit the building at any time. The pathway logic dictates that population groups move along the shortest, most logical path between assigned zones.

### 4.5 Office workday scenario

**Fig 8:**
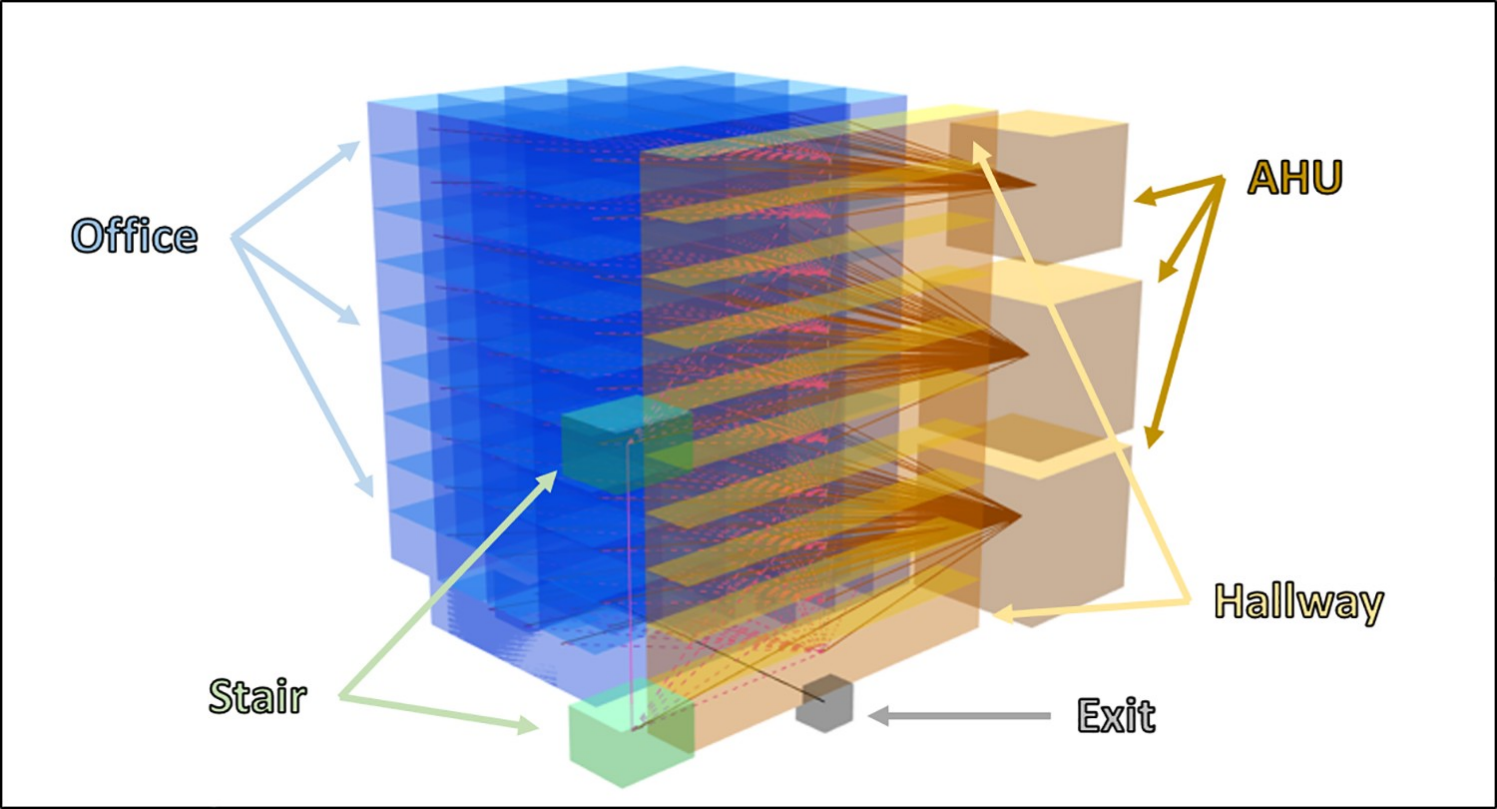
The simulated office building comprises individual offices connected by shared hallways, staircases, and air handling units (AHU).

We modeled an office building consisting of 10 floors, each about 10,750 square feet in area, roughly similar to the Key Center South Tower building in Buffalo, NY [67]. The simulated building had three large Air Handling Units (AHU), each servicing multiple floors. The default value for ACH of 4–10 was taken from Arthur Bell’s HVAC Equations, Data, and Rules of Thumb [46]. Additional model parameters are given in Table 10. Each floor was divided into a hallway and 20 connected offices of about 460 sq. ft. each. In this way, each office represented approximately two to four standard individual offices. Two other zones representing staircases connecting the floors were also modeled. Though the model uses a network-zone concept, a visualization of this building layout is shown in Fig 8; the location and shapes of the zones are notional.

**Table 10:** Office Model Parameters.

Parameter	Value
**Number of floors**	10
**Floor Size**	10,750 sq. ft.
**Number of AHUs**	3
**ACH**	4–10 1/hour
**Office zone size**	460 sq. ft.
**Population**	1082 persons
**Group Size**	6 persons/group
**Residence Time**	~8 hours

*SARS-CoV-2 was released in an office on the 5*^*th*^ *floor of the building*, *representing the zone with an infected individual*. As previously mentioned, the office was modeled with a population of 1082 persons. Each population group in the office building comprised approximately six persons, yielding approximately 170 to 180 groups in each simulation. Each of these groups were assigned different movement schedules to fill a workday lasting approximately eight hours. For each group, a random number of meetings were assigned, with each meeting lasting some multiple of half-hours and scheduled to begin on the hour or half-hour. These meetings would be held in randomly selected office zones throughout the building. At the appropriate scheduled time, the groups would move to that location to attend the meeting, and stay there for the duration of the meeting, returning to their own offices after the meeting had finished, or moving onward to the next meeting. Each group was also given a probability to take a 30- to 60-minute lunch break in the middle of the day. Groups taking a lunch break would exit the building on the ground floor and would not receive exposure while outside the building. They would begin receiving exposure again after re-entering the building following their break. Groups that were assigned zero meetings and who were not scheduled to take a lunch break would occupy their own office zones for the entire duration of their residence in the model, only passing through other zones as they entered or left the building. The model simulation ends when all groups at the close of business day have exited the building.

### 4.6 Social events scenarios

Social gatherings of large or moderately sized groups of people in a confined indoor environment were also investigated as part of this effort. The efficacy of HVAC systems at mitigating or spreading SARS-CoV-2 was evaluated for social gatherings taking place in single-story buildings with limited compartmentalization. The three types of single-floor social events that were modeled are:

- Bar/Restaurant
- Nightclub
- Wedding Reception

**Fig 9:**
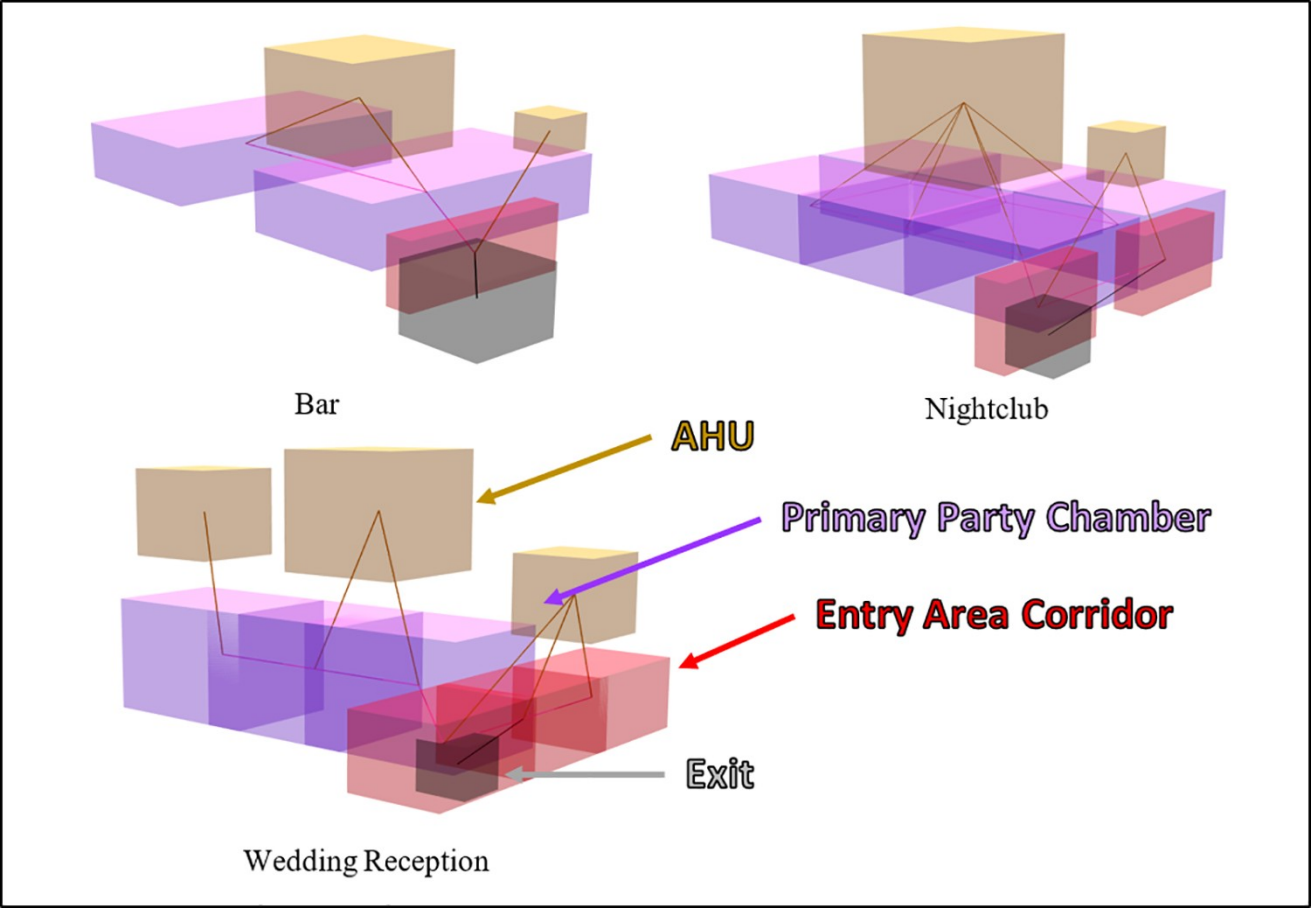
Social gathering building layouts.

Each event was modeled using a building consisting of a large open chamber area and a smaller corridor area. The larger open area represented the seating and eating/drinking area where people would spend most of their time. The smaller corridor area was where people would enter and exit the building and represented a reception/lobby area and where the restrooms would be located. Table 11 shows the sizes of the buildings used for each event. Fig 9 shows the layouts of the three single-floor, social gathering event types. For each event, one of the chamber zones was randomly selected for the location of the SARS-CoV-2 release.

**Table 11:** Social gathering building sizes and ACH.

Venue	Specific Building Used	Area [ft^2^]	Ceiling Height [ft]	ASHRAE Standard 6.2 Building Type	Default ACH
**Bar/Restaurant**	Unspecified restaurant, Washington D.C.	1,400	10	Food and Beverage Service	11.1
**Nightclub**	Unspecified nightclub [68]	7,000	10	Disco/dance floor	19.8
**Wedding Reception**	Unspecified Catering Hall, Washington D.C.	3,600	30	Public Assembly Space	2.48

For each of the three events, population sizes were modeled using discrete uniform distributions representing high, medium, and low populations for the venue. The different population sizes were used to represent different degrees of social distancing, but no analysis is presented in this report on the specific impact of population density on the infection risk. Residence times for the groups were modeled using uniform distributions. Total population would be drawn once per model realization. Residence time was drawn once per modeled group, and Table 12 shows the low, medium, and high population sizes modeled for each event and the residence times of the modeled population groups.

**Table 12:** Social gathering population size and residence time.

Venue	Population (uniform discrete)	Residence Time [min] (uniform)
**Bar/Restaurant**	10, 35, 70	45–90
**Nightclub**	50, 250, 500	120–300
**Wedding Reception**	50, 100, 225	180–300

Each group in the social event models receives a basic pattern of arrival time, residence in a particular zone, and exit. The proximity of each group to the infected individual was limited to the zone of their residence. It was assumed that the confined space and lack of certainty of the movement patterns of the infected individual (who is also assumed to not move) made modeling group behavior patterns unlikely to result in realistic movements. The zone of residence, then, is meant to represent the zone in which the population group spent the majority of its time.

Default ACH values were developed using ASHRAE standards for indoor air quality based on expected occupancy, area of indoor space, and type of activity for which the space is intended. ***Equation 5*** from ASHRAE Standard 62 [38] was used to determine the necessary outside air rate.

**Equation 5**: Required outside air rate

$$R_{o}=R_{A}*A+R_{P}*P$$

Where:

- R_o_−Outside Air Rate in cubic feet of air per minute
- R_A_−Area Outdoor Air Rate in cubic feet of air per minute per square meter of building area
- R_P_−People Outdoor Air Rate in cubic feet of air per minute per person
- A–Area of the Building in square feet
- P–Expected Maximum Population of the Building

Values for R_A_ and R_P_ were also taken from ASHRAE Standard 62 for the comparable ASHRAE building type (see Table 11). ACH was then calculated using the outside air rate and the approximate interior volume of the building and FOA, with FOA set to the expected value from the outside air fraction distribution of ~0.3. Final default values used are also listed in Table 11.

**Equation 6**: Social gathering ACH

$$ACH=60*\frac{R_{o}}{FOA}*\frac{1}{V}$$

Where:

- ACH–Air Changes per Hour
- FOA–The fraction of outside air, assumed to be ~0.3 for the default ACH estimate
- V–The approximated total volume of the building in cubic feet

## Supporting information

S1 Text Additional review of UVC efficacy, analysis of proximity to release zone on infection risk, and validation of model results against South Korean apartment complex outbreak. (DOCX)

S1 Data Model results underlying Figs 1–5 and Tables 2 and 3. (ZIP)
