# Supplementary material for: Factors affecting aerosol SARS-CoV-2 transmission via HVAC systems; a modeling study
Source: PLoS Comput Biol. 2021 Oct 18;17(10):e1009474. doi: 10.1371/journal.pcbi.1009474 (PMC8553169; doi:10.1371/journal.pcbi.1009474)
Supplement: S1 Text — (DOCX) [file pcbi.1009474.s001.docx]

Title: Factors affecting aerosol SARS-CoV-2 transmission via HVAC systems; a modeling study

Authors: Zachary J. Cotman^1^, Michael J. Bowden^1^, Barrett P. Richter^1^, Joseph H. Phelps^1^, and Christopher J. Dibble^1,*^

Affiliations: ^1^Battelle Memorial Institute, Columbus, Ohio

^*^Corresponding Author

E-mail: [dibble@battelle.org](mailto:dibble@battelle.org)

Orcid: <https://orcid.org/0000-0002-0303-7330>

# Appendix A

### UVC decontamination in HVAC systems

UVC devices have been widely investigated for HVAC decontamination in the academic literature, though two primary references are useful when assessing UVC decontamination for human coronaviruses. Walker and Ko (2007) used an experimental chamber to estimate the dose-decontamination curve for murine hepatitis virus (MHV), a coronavirus commonly used as a surrogate for human pathogens [1]. In this study, MHV was aerosolized via Collison nebulizer and drawn through the chamber at a rate of 12.5 L/min, with virus passing by a UVC (254 nm) light before deposition in an aerosol sampler [1]. The researchers found that a dose of 599 μW * s/cm^2^ (μW is microwatts), or approximately 0.66 mJ/cm^2^ was enough to inactivate 88% of aerosolized MHV within a 16.2-second contact time [1]. Similarly, Buonanno et al. (2020) used UVC at 222nm to inactivate two human coronaviruses (229-E and OC43) in an experimental chamber [2]. In this study, a dose of 0.56 mJ/cm^2^ (for 229-E) or 0.39 mJ/cm^2^ (for OC43) inactivated 90% of aerosolized particles with a 20-second contact time [2]. S1 Table 1 provides a summary of other estimates of UVC inactivation rates for aerosolized viruses, alongside a qualitative estimate or relevance for SARS-CoV-2. In general, low-dose UVC appears capable of inactivating coronaviruses, though experimental estimates for SARS-CoV-2 were not identified in the review.

Data on dose-inactivation in residential or commercial HVAC systems were also identified in the literature review, though information comparable to academic studies was sparse. However, commercial systems from Fresh-Aire report both the exposure dose and efficiency (% reduction) of different units for an unspecified coronavirus. For instance, a 5-ton commercial HVAC model generated an exposure dose of 5.1 mJ/cm^2^ (Millijoules/cm^2^) with a 2,000 CFM (cubic feet per minute) airflow rate and a 0.2 second exposure time. This was enough to inactivate >99.99% of aerosolized coronavirus, though some specific details (e.g., specific virus, sampling method, quantification method) are not provided [3]. Nevertheless, 5 mJ/cm^2^ of total exposure is an order of magnitude higher than the 0.4 – 0.7 mJ/cm^2^ doses required to inactivate 90% of coronaviruses in Walker and Ko (2007) and Buonanno et al. (2020), suggesting that a 90% minimum reduction is plausible with commercial systems. Comparable data for residential systems was not identified, though American Ultraviolet reports doses of 11.7 μW/cm^2^ for their product [4]. This dose is larger than the dose required to inactivate 80% of aerosolized tuberculosis bacteria (6 μW/cm^2^) in Mphaphlele et al., 2015, [5] which suggests some ability to inactivate viruses in home UVC systems, given that bacteria are generally more resistant to UV damage than viruses.

From this limited review, it appears that commercial HVAC systems, and perhaps residential systems, are capable of delivering UVC doses sufficient to inactivate at least 90% of aerosolized coronaviruses. Manufacturer data supports inactivation efficiencies up to 99.99% for commercial systems. It is suggested that a range of inactivation efficiencies from 90% to 99.9% be utilized in subsequent modeling work.

**S1 Table 1**. Summary of UVC dose-inactivation estimates for aerosolized viruses, with qualitative indication of relevance to the current effort.

| Relevance | Agent type | Agent | Wavelength (nm) | Dose (mJ/cm^2^) | Efficiency  (% reduction in active agent) | Reference |
| --- | --- | --- | --- | --- | --- | --- |
| High | Mouse coronavirus | MHV | 254 | 0.599 | 88 | Walker and Ko, 2007 [1] |
| High | Human coronavirus | HCoV-229E | 222 | 0.56 | 90 | Buonanno et al., 2020 [2] |
| High | Human coronavirus | HCoV-229E | 222 | 1.7 | 99.9 | Buonanno et al., 2020 [2] |
| High | Human coronavirus | HCoV-OC43 | 222 | 0.39 | 90 | Buonanno et al., 2020 [2] |
| High | Human coronavirus | HCoV-OC43 | 222 | 1.2 | 99.9 | Buonanno et al., 2020 [2] |
| Medium-High | Mouse coronavirus | MHV | 254 | 0.66 | 90 | Hebling et al., 2020 [6] |
| Medium-High | Human influenza | H1N1 | 222 | 1.6 | 95 | Welch et al., 2018 [7] |
| Medium-High | Human influenza | H1N1 | 254 | 1.1 | NS | Welch et al., 2018 [7] |
| Medium-High | Mouse coronavirus | MHV | 254 | 0.599 | 88 | Welch et al., 2018 [7] |
| Medium-High | Human influenza | H1N1 | 254 | 1.05 | 90 | McDevitt et al., 2012 [8] |
| Medium-High | Human influenza | H1N1 | 254 | 0.85 | 90 | McDevitt et al., 2012 [8] |
| Medium-High | Human influenza | H1N1 | 254 | 0.79 | 90 | McDevitt et al., 2012 [8] |
| Medium-Low | Phage | MS2 | 254 | 46 | 0.999980047 | Kim and Kang, 2018 [9] |
| Medium-Low | Phage | PhiX174 | 254 | 4.6 | 0.9999 | Kim and Kang, 2018 [9] |
| Medium-Low | Phage | Qbeta | 254 | 46 | 0.999987411 | Kim and Kang, 2018 [9] |
| Medium-Low | Human coronavirus | SARS-CoV-2 | 254 | 0.3 | 37 | Sagripanti and Lytle, 2020 [10] |
| Medium-Low | Human coronavirus | Coronavirus | 254 | 0.61 | 90 | Brais 2019 [11] |
| Medium-Low | Phage | dsDNA (T7) | 254 | 1.053 | 90 | Tseng and Li, 2007 [12] |
| Medium-Low | Phage | dsRNA (phi 6) | 254 | 0.7625 | 90 | Tseng and Li, 2007 [12] |
| Medium-Low | Phage | ssDNA (phi X174) | 254 | 0.469 | 90 | Tseng and Li, 2007 [12] |
| Medium-Low | Phage | ssRNA (MS2) | 254 | 0.381 | 90 | Tseng and Li, 2007 [12] |
| Low | Human coronavirus | coronaviruses | 254 | 0.611 | 90 | UVDI, 2020 [13] |
| Low | Human influenza | H1N1 | 254 | 1.935 | 90 | UVDI, 2020 [13] |
| Low | Human coronavirus | coronaviruses | <280 | <1.0 | 90 | UV Clinical, 2020 [14] |

*NS = wavelength not specified. Reference indicates that the UV source was a General Electric lamp number G36T6.*(15)

# Appendix B

## Evaluation of Infected Groups with Respect to Release Zone Proximity

A detailed look at infections by group and the proximity of the groups to the release zone in the model was performed to understand which modeled persons were being infected. Analysis was performed for the office building and nightclub scenarios. S1 Table 2 shows the release rates and HVAC parameter settings used for this investigation. Permutations of all possible MERV rating, FOA, and ACH setting were not evaluated, but rather collections of low, medium, and high HVAC setting were used.

**S1 Table 2**. Parameters Used to Investigate Infections by Group and Proximity to Release

| Release Rates (PFU / min) | HVAC Setting | | | | | |
| --- | --- | --- | --- | --- | --- | --- |
| 100 | Low | | Medium | | High | |
| 1,000 | MERV | 4 | MERV | 8 | MERV | 12 |
| 3,000 | ACH | 2 | ACH | 10 | ACH | 20 |
| 10,000 | FOA | 0.1 | FOA | 0.3 | FOA | 0.5 |

Because group dynamics change slightly on a per-model-realization basis and the goal was to evaluate individual group infections, only a single realization was used for each combination of target, release rate, and HVAC setting. To capture the most likely scenario with a single realization, mean values for the modeled decay rate and SARS-CoV-2 particle size were used rather than the distributions.

In general, results show that groups spending a lot of time in the release zone have a much higher rate of infection than those outside the release zone. As HVAC parameters increase from low to high, infections in zones other than the release zone decrease to zero, while groups spending significant time in the release zone continue to be infected for release rates of 1,000 PFU / min and up. For the release rate of 100 PFU / min, no scenario resulted in infections.

### Office Scenario Infections by Group and Proximity to Release Zone

S1 Table 3 shows infections for all infected groups in the Office scenario with a release rate of 10,000 PFU / min and low HVAC settings. This scenario was selected as it demonstrates well the relation between a group’s proximity to the release zone and resulting infections. A total of 180 groups were modeled. Groups not shown in S1 Table 3 received no infections.

**S1 Table 3**. Group Infections for Office with Release Rate of 10,000 PFU / min and Low HVAC Settings

| ID | Size | Infections | Time in Release Zone (min) | Time on Release Floor (min) | Total Time in Building (min) | AHU Servicing Primary Residence Zone |
| --- | --- | --- | --- | --- | --- | --- |
| 81 | 6 | 6 | 445 | 451 | 515 | 1 |
| 11 | 6 | 6 | 116 | 120 | 546 | 1 |
| 87 | 6 | 1 | 0 | 505 | 509 | 1 |
| 99 | 6 | 1 | 0 | 520 | 524 | 1 |
| 97 | 6 | 1 | 0 | 524 | 531 | 1 |
| 92 | 6 | 1 | 0 | 478 | 544 | 1 |
| 95 | 6 | 1 | 0 | 499 | 506 | 1 |
| 23 | 6 | 1 | 0 | 0 | 555 | 1 |
| 83 | 6 | 1 | 0 | 452 | 519 | 1 |
| 78 | 6 | 1 | 0 | 0 | 532 | 1 |
| 98 | 6 | 1 | 0 | 513 | 520 | 1 |
| 82 | 6 | 1 | 0 | 447 | 514 | 1 |
| 94 | 6 | 1 | 0 | 474 | 486 | 1 |

All individuals from both groups (groups 81 and 11) that visited the release zone were infected. No other group visited the release zone. Most groups with some infections spent significant time on the same floor as the release zone. The two groups that did not spend time on the same floor as the release (groups 23 and 78) spent most of their time on a floor serviced by the same air handling unit (AHU) and were in the top 10^th^ percentile for total time spent in the building. S1 Fig 1 shows that most infected individuals visited the release zone during the workday. There were two AHUs in this simulation, one servicing floors 0-5, and the other servicing floors 6-9.


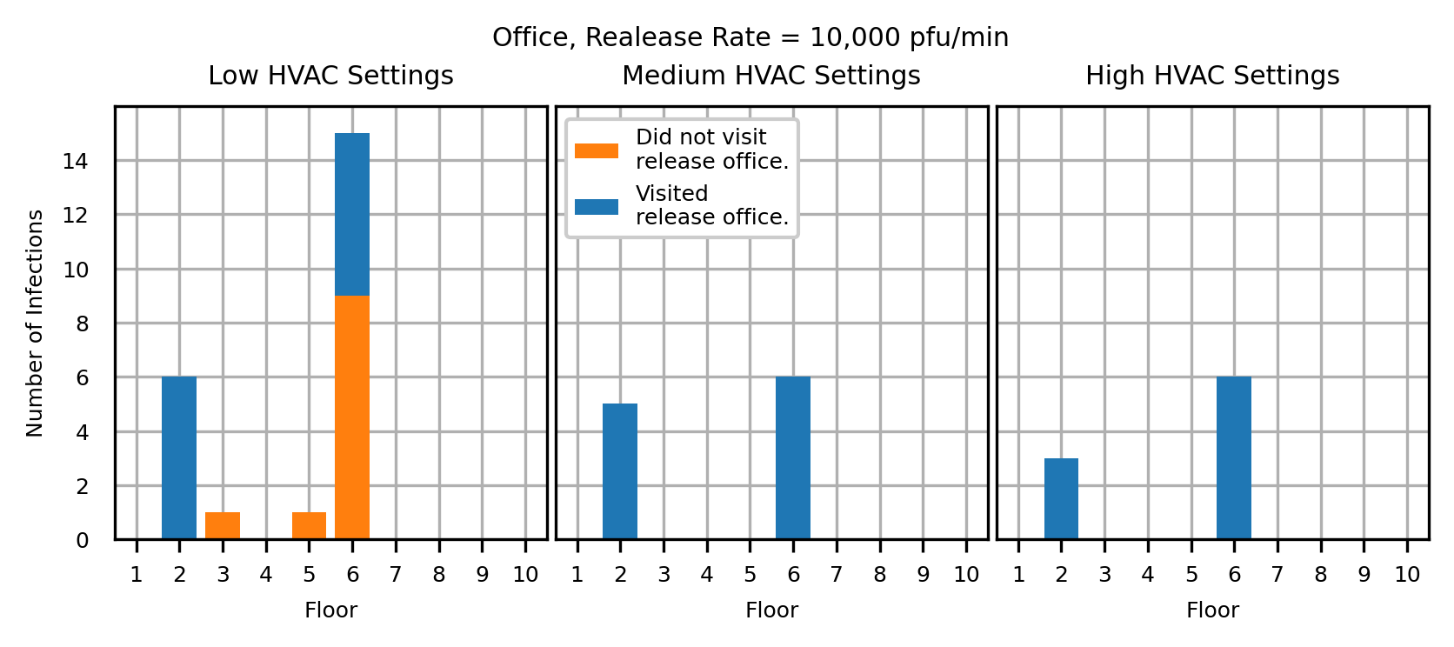


**S1 Fig 1**. Number of infections by floor for all groups not visiting the release zone for an office scenario with a release rate of 10,000 PFU / min and low HVAC settings.

For all other office building scenarios with release rates below 10,000 PFU / min or medium and high HVAC parameter settings, the only groups receiving infections were the two groups that visited the release zone: groups 81 and 11. As previously mentioned, there were no injuries for any group for a release rate of 100 PFU / min. S1 Table 4 shows the infections for groups 81 and 11.

**S1 Table 4**. Infections for Groups in Office Building Scenario That Spent Time in the Release Zone

| **HVAC Settings** | **10,000 PFU / min** | | **3,000 PFU / min** | | **1,000 PFU / min** | |
| --- | --- | --- | --- | --- | --- | --- |
|  | **Group 81** | **Group 11** | **Group 81** | **Group 11** | **Group 81** | **Group 11** |
| Low | 6 | 6 | 6 | 5 | 5 | 2 |
| Medium | 6 | 5 | 5 | 2 | 2 | 0 |
| High | 6 | 3 | 3 | 1 | 1 | 0 |

### Nightclub Scenario Infections by Group and Proximity to Release Zone

Like the office building scenario, groups in the release zone for the office scenario received the most infections. Unlike the office scenario, some groups outside the release zone received infections for 10,000 PFU / min with medium HVAC settings and 3,000 PFU / min with low HVAC settings. S1 Fig 2 shows infections by zone for a 10,000 PFU / min release rate and all three HVAC settings: low, medium, and high. Zone 4 is the release zone. The six nightclub chamber zones were the primary residence zones for all groups, so no corridor zones are represented in S1 Fig 2.


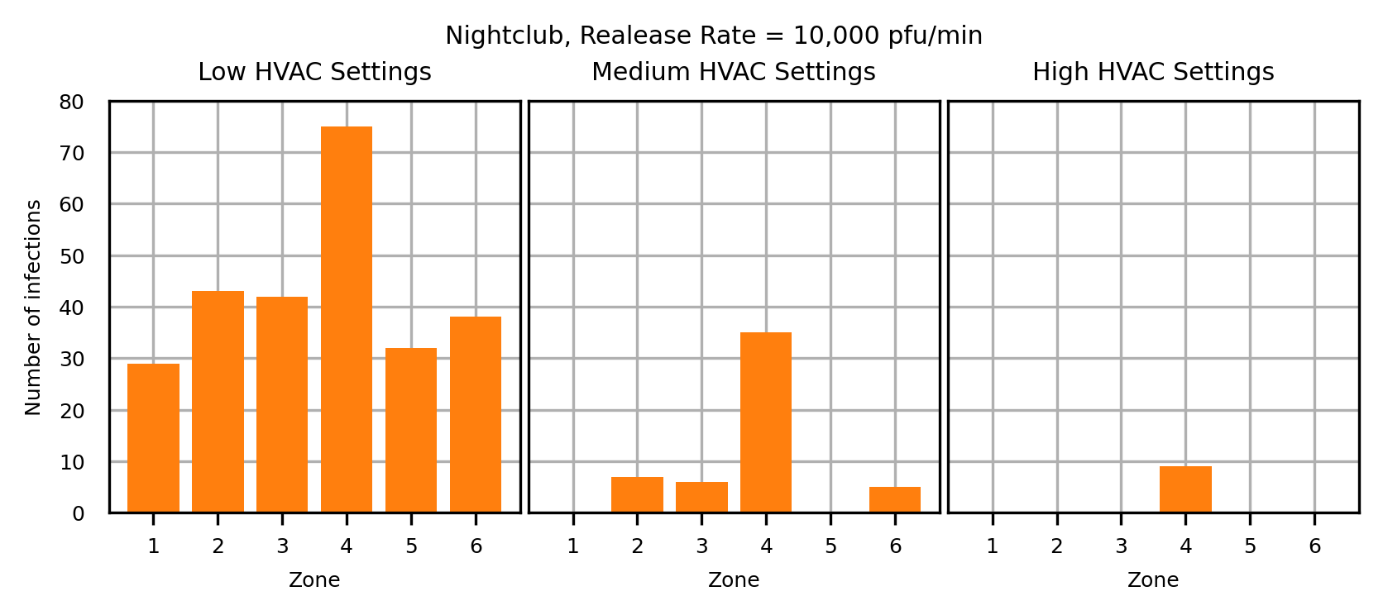


**S1 Fig 2**. Nightclub infections by chamber zone for a release of 10,000 PFU / min using Low, Medium, and High HVAC Settings. Zone 4 is the release zone.

For the nightclub scenario, groups in zones near the release zone are more likely to be infected than for the office scenario (S1 Table 5). The office zones are modeled with door connections that have slower rates of air exchange than the open connections between the chamber zones in the nightclub scenario. For higher release rates and lower HVAC parameters, this mechanic allows for more infections outside the release zone. Conversely, for lower release rates and higher HVAC settings, the increased rate of dispersion results in fewer total infections, as the concentration in the release zone drops faster in the open chamber release zone than in the office room release. This converse effect can be seen by comparing release zone infections for office and nightclub shown in S1 Fig 1 and S1 Fig 2 respectively; the relative decrease in infections for the high HVAC settings is much greater for the nightclub than for the office. The higher number of infections in the nightclub release zone for high release rates and lower HVAC settings is due to the high nightclub population density.

**S1 Table 5**. Infections in the Release Zone for All Nightclub Scenarios

| HVAC Settings | Release Rate | | |
| --- | --- | --- | --- |
|  | **10,000 PFU / min** | **3,000 PFU / min** | **1,000 PFU / min** |
| Low | 100 | 40 | 6 |
| Medium | 38 | 4 | 0 |
| High | 9 | 0 | 0 |

# Appendix C: Validation with South Korean Apartment Complex Outbreak

Hwang et. al. describes the scenario where cases of SARS-CoV-2 infection may be linked to a shared vertical ventilation shaft connecting bathrooms in an apartment complex in South Korea [16]. A simple mockup of the scenario was created using our indoor transport and infection model for comparison.

## Infection Event

In the event, a total of 10 people became infected in the apartment complex along 2 adjoined sets of 13 vertically-stacked apartments, designated “Line A” and “Line B” in the paper. Each set had a shared ventilation shaft connecting to the apartments via vent openings in the bathrooms.

Diagnosis of all patients occurred between the 23^rd^ and 27^th^ of August 2020 with reported dates of first symptoms between the 16^th^ to 24^th^. All patients reportedly had no contact or relation besides living in the same apartment complex. Of the 10 patients diagnosed with SARS-CoV-2, 8 lived in apartments on Line A, and 2 in apartments on Line B. As well, the patient with the earliest symptoms resided in an apartment on Line A.

In the paper, the authors identify the ventilation shaft as a probable cause of spread as it was the only open connection between the apartments, and the patients reported having worn masks when outside their own apartments, decreasing the likelihood of droplet spread in shared hallways, elevators, and staircases in the building. A key factor in the ventilation shaft is the lack of backdraft dampers and even a lack of fan units for most of the apartments. Fan units, when on, would force air out of the bathroom into the shaft. The authors describe stack effect as a primary driver of air transmission between the apartments, shaft, and outside.

## Simulated Scenario

In the description of the event, there seems to be no direct connection between apartments on Line A and its ventilation shaft and Line B. As well, the majority of the infections (8 out of 10) occurred along a single line: Line A. For this reason, we limited the model to a single line with 13 building zones modeled to represent the 13 apartments. Each was given its own mechanical HVAC system. A network of small connections was created between all 13 zones to represent the shared natural ventilation shaft. Each vent opening was modeled as $0.1 m^{2}$ ($\sim1 ft^{2}$) in area. Since this opening was modeled as the connection via which a single apartment connects to all other apartments, each connection was reduced by a factor of 1/12^th^, that is, $1/{(n_{apartments}-1)}$. It was over these connections that air was allowed to travel between the apartments. Recall that the model applies bi-directional air flow via simple volume exchange between connected zones at each time step. This is a limitation of the model as it does not simulate stack effect, which is the flow of air due to temperature-induced pressure gradients along the vertical shaft.

A subset of HVAC parameters was chosen to represent a range of possibilities that may exist in a residential setting. Settings for a specific realization were chosen assuming a uniform distribution across these ranges. Some combinations of low settings would be representative of systems that do not meet residential ASHRAE standards, while other combinations of high settings represent systems far exceeding these standards [17]. Indoor air speed, which dictates how much air flows across a modeled connection in a single time step, was drawn from a range of 0.05-0.3 $m/s$ which is slightly expanded from indoor air speeds generally considered to be “comfortable” and not drafty [18]. The actual flow rates between the connected bathrooms and vertical vent shaft are unknown. MERV filter rating was varied systematically from poor to excellent for residential settings.

Apartment size was not specified in the reported event. We chose $1,200 ft^{2}$ as a reasonable size which a small family might inhabit. The paper reported “437 residents from 267 households” as being tested across the whole apartment complex. This gives approximately 1.6 residents per apartment, which we assumed in the model.

We did not simulate multiple stages of spread of SARS-CoV-2, but rather assessed the possibility of secondary infections from the initial infected spreader. To do this, we assumed a constant release rate from the spreader in a single apartment and used the model to determine the probability of infection in a single connected apartment. This was multiplied by the 19 expected people in the 12 other connected apartments to determine the expected number of resulting infections.

We simulated a symptomatic release occurring over a 3 day period starting with a building free of aerosols. The model determines an extra amount of time to run by calculating how long it will take for SARS-CoV-2 to decay through biological processes to 1/10,000^th^ of the total release amount. This overshoots the time necessary to clear the building of aerosols as they will also be continually settling, filtered, and vented. Simulated time was approximately 3.7 days. Release rates selected for this validation were also systematically varied.

Finally, the total resulting doses received by a person residing in a connected apartment were multiplied by 1, 2/3^rd^, and 1/3^rd^ to estimate the approximate dose experienced by a person who spends on average, 24 hours, 16 hours or 8 hours a day in their apartment.

The same parameters were used for SARS-CoV-2 probit slope, ID_50_, particle sizes, and decay as used for other modeling (see Table 1 of the manuscript). S1 Table 6 summarizes other parameters used in this validation study.

**S1 Table 6**. Parameters used in validation modeling of a 3-day release in connected, multistory apartments

| Parameter | Value or Range |
| --- | --- |
| ACH | 1 - 4 |
| FOA | 0.1 - 0.3 |
| MERV Filter Rating | 4, 8, 12 |
| Indoor Air Speed | 0.05 - 0.3 $m/s$ |
| Number of Apartments | 13 |
| Apartment Size | 1,200 $ft^{2}$ |
| Shared vent opening | 0.1 $m^{2}$ |
| Average number of residents per apartment | 1.6 |
| Simulated Release Time | 3 days |
| Total Simulated Time | 3.7 days |
| Release Rates | 100, 500, 1000, 2000, 3000 $PFU/min$ |
| Time spent in connected apartment | 8, 16, 24 ${hours}/{day}$ |

## Results

**S1 Table 7**. Min, Median, and Max predicted infections from simulated 3 day release in apartments connected in a multistory complex.

| Release Rate PFU/min | Predicted number of infections out of 19 exposed | | |
| --- | --- | --- | --- |
|  | Min | Median | Max |
| 100 | 0.015 | 0.104 | 0.528 |
| 500 | 0.177 | 0.787 | 2.564 |
| 1000 | 0.429 | 1.577 | 4.282 |
| 2000 | 0.933 | 2.853 | 6.512 |
| 3000 | 1.399 | 3.854 | 7.989 |

As can be seen in S1 Table 7, the model predicts reasonable possibilities of infections, even with release rates which are not extremely high. It does not predict that all residents become infected from a single initial spreader or that very few (much less than 1) become infected, except for some realizations with the lowest release rate of 100 PFU/min. If the model predicted that all residents would be infected, or if it predicted much fewer than 1 infections for all scenarios, this test would invalidate the methods used, and correction of said methods would be necessary.

Again, many aspects of release and infection from SARS-CoV-2, such as actual release rates and median infectious doses remain key data gaps. Many of the exact parameters for the apartment scenario such as actual HVAC performance and apartment size were also unknown. As well, the model does not account for subsequent compound exposures due to secondary infections. That is, in the real event, early secondary infections would have contributed to exposure for any resident not yet infected, possibly increasing their probability of infection. As previously stated, this multi-stage infection was not modeled.

Interestingly, the spread in results seems to indicate that HVAC performance may have more potential impact in residential settings where exposure duration is longer and standards for required fresh air are lower. These possibilities were not explored further in this study.

# References

1. Walker CM, Ko G. Effect of Ultraviolet Germicidal Irradiation on Viral Aerosols. Environmental Science & Technology. 2007;41(15):5460-5.

2. Buonanno M, Welch D, Shuryak I, Brenner DJ. Far-UVC light (222 nm) efficiently and safely inactivates airborne human coronaviruses. Scientific Reports. 2020;10(1):10285.

3. Fresh-Aire. Air Disinfection Analysis - Report. 2020.

4. Ultraviolet A. Duct Clean (DC) Series. American Ultraviolet.

5. Mphaphlele M, Dharmadhikari AS, Jensen PA, Rudnick SN, Reenen THv, Pagano MA, et al. Institutional Tuberculosis Transmission. Controlled Trial of Upper Room Ultraviolet Air Disinfection: A Basis for New Dosing Guidelines. American Journal of Respiratory and Critical Care Medicine. 2015;192(4):477-84.

6. Heßling M, Hönes K, Vatter P, Lingenfelder C. Ultraviolet irradiation doses for coronavirus inactivation–review and analysis of coronavirus photoinactivation studies. GMS hygiene and infection control. 2020;15.

7. Welch D, Buonanno M, Grilj V, Shuryak I, Crickmore C, Bigelow AW, et al. Far-UVC light: A new tool to control the spread of airborne-mediated microbial diseases. Scientific Reports. 2018;8(1):2752.

8. McDevitt JJ, Rudnick SN, Radonovich LJ. Aerosol susceptibility of influenza virus to UV-C light. Appl Environ Microbiol. 2012;78(6):1666-9.

9. Kim D-K, Kang D-H. UVC LED Irradiation Effectively Inactivates Aerosolized Viruses, Bacteria, and Fungi in a Chamber-Type Air Disinfection System. Applied and Environmental Microbiology. 2018;84(17):e00944-18.

10. Sagripanti J-L, Lytle CD. Estimated Inactivation of Coronaviruses by Solar Radiation With Special Reference to COVID-19. Photochemistry and Photobiology. 2020;96(4):731-7.

11. Brais NP. Air Disinfection for ART Clinics using Ultraviolet Germicidal Irradiation. preprint. 2020.

12. Tseng C-C, Li C-S. Inactivation of Virus-Containing Aerosols by Ultraviolet Germicidal Irradiation. Aerosol Science and Technology. 2005;39(12):1136-42.

13. UVDI. Effectiveness of UVC Light to Mitigate Coronavirus (COVID-19). 2020.

14. Clinical U. The germicidal power of UVC light against coronavirus 2020 [Available from: <http://uvclinical.com/2020/04/06/the-germicidal-power-of-uvc-light-against-coronavirus/>.

15. Jensen MM. Inactivation of airborne viruses by ultraviolet irradiation. Applied microbiology. 1964;12(5):418-20.

16. Hwang SE, Chang JH, Oh B, Heo J. Possible aerosol transmission of COVID-19 associated with an outbreak in an apartment in Seoul, South Korea, 2020. International Journal of Infectious Diseases. 2021;104:73-6.

17. American Society of Heating R, and Air-Conditioning Engineers. ASHRAE Standard 62.2-2019, Ventilation for Acceptable Indoor Air Quality in Residential Buildings. Atlanta, GA2019.

18. American Society of Heating R, and Air-Conditioning Engineers. ASHRAE Standard 55-2020, Thermal Environmental Conditions for Human Occupancy. Atlanta, GA2020.
